# The Influence of Motor Competence on Broader Aspects of Health: A Systematic Review of the Longitudinal Associations Between Motor Competence and Cognitive and Social-Emotional Outcomes

**DOI:** 10.1007/s40279-023-01939-5

**Published:** 2023-11-21

**Authors:** Phillip J. Hill, Melitta A. Mcnarry, Kelly A. Mackintosh, Maeve Aine Murray, Caterina Pesce, Nadia C. Valentini, Nancy Getchell, Phillip D. Tomporowski, Leah E. Robinson, Lisa M. Barnett

**Affiliations:** 1 Applied Sports, Technology, Exercise and Medicine (A-STEM) Research Centre, Swansea University, Swansea, SA1 8EN Wales UK; 2 School of Health and Human Performance, Dublin City University, Dublin, D09 F8Y6 Ireland; 3 Department of Movement, Human and Health Sciences, University of Rome “Foro Italico”, Rome, Italy; 4 Universidade Federal do Rio Grande do Sul, Porto Alegre, Brazil; 5 Developmental Motor Control Laboratory, 157 Human Performance Lab, University of Delaware, 540 S College Ave, Newark, 19713 DE UK; 6 Department of Kinesiology, University of Georgia, Athens, GA USA; 7 School of Kinesiology, University of Michigan, SKB 1054; 830 North University, Ann Arbor, MI 48109-1048 USA; 8 Institute for Physical Activity and Nutrition, Faculty of Health, School of Health and Social Development, Deakin University, Building BC, 221 Burwood Hwy, Burwood, Melbourne, 3125 Australia

## Abstract

**Background:**

Motor competence has important developmental associations with aspects of physical health, but there has been no synthesis of longitudinal associations with cognitive and social-emotional health.

**Objectives:**

The first aim was to present a conceptual model that positions motor competence as a mediator between physical activity and cognitive and social-emotional outcomes. The second aim was to synthesize the association of motor competence and cognitive and social-emotional development using longitudinal observational and experimental evidence, in particular to (i) identify the role of task, individual, and environmental characteristics in moderating the association between motor and cognitive and social-emotional outcomes and (ii) synthesize the strength of evidence pertaining to domain-specific relationships.

**Methods:**

This systematic review was registered with the International Prospective Register of Systematic Reviews (PROSPERO) and adhered to the Preferred Reporting Items for Systematic Reviews and Meta-Analyses (PRISMA) statement for reporting systematic reviews and meta-analyses. Five electronic databases (PubMed, Web of Science, Scopus, PsycINFO, and SPORTDiscus) were systematically searched. Following study screening and risk-of-bias assessment by two authors, 49 eligible studies were identified for inclusion and grouped by study design. Evidence for domain-specific paths between motor competence and cognitive and social-emotional outcomes was synthesized by calculating the significant analyses in the hypothesized direction, divided by the total number of analyses for that path. These percentages were then collated for each domain outcome. This collated influence was classified as either no association (0–33%), written as ‘0’, or indeterminate/inconsistent (34–59%), written as ‘?’ If there were fewer than three studies in the domain, the strength of evidence was classified as insufficient (I).

**Results:**

Of the 49 studies, 35% were able to satisfy six or more of the seven risk-of-bias criteria. Longitudinal observational evidence about domain-specific and global associations of motor competence and cognitive and social-emotional development is indeterminate. The included studies also did not provide evidence for a consistent moderating role of age and sex. Some preliminary experimental evidence does support the role of motor competence in moderating the influence of cognitively enriched physical activity on cognitive outcomes, especially working memory and social-emotional skills. However, too few studies were appropriately designed to acknowledge the moderating role of contextual mechanisms.

**Conclusions:**

Between-study heterogeneity means it was not possible to identify definitive domain- and construct-specific relationships between motor competence and cognitive and social-emotional outcomes. To further develop our understanding, it is important that researchers acknowledge the complexity of these relationships within rigorous study designs.

**Supplementary Information:**

The online version contains supplementary material available at 10.1007/s40279-023-01939-5.

## Key Points

**Table:** 

Our review presents indeterminate observational evidence supporting the influence of motor competence on aspects of executive functions and academic performance, with clear patterns of domain-specific relationships not manifest. Similarly, the included experimental evidence only offers preliminary support for the alignment between the underlying processes responsible for executive functions (e.g., working memory) and those deemed important for engaging in enriched movement interventions.
Many studies were lacking in methodological rigor, and failed to sufficiently report on the moderating and contextual factors that may, or may not, trigger mechanisms acting in the relationship between physical activity, motor competence, and cognitive and social-emotional outcomes.
Experimental studies need to prioritize the inclusion of thorough process evaluations, providing researchers the opportunity to consistently identify those characteristics of an intervention that may prompt a causal or moderating influence.

## Background

Motor competence refers to the goal-directed and coordinated motor acts (e.g., running and throwing) that provide the basis for the complex movement patterns required for various physical activity contexts and participation in many sports [1]. Assessment of motor competence primarily adheres to either a process or product-oriented approach, with these providing insight into the quality and/or outcome of specified movements [2, 3]. Assessment is commonly performed one skill at a time but more recently circuit-based assessment with linked skills (which can involve product and occasionally process approaches) have been used. The underlying rationale of the assessment and the context in which it is being delivered mean many assessment methods exist [4].

In 2008, Stodden and colleagues [5] proposed a conceptual model to illustrate the critical role of motor competence in developing positive and negative health trajectories during childhood. Central to the model authored by Stodden et al. [5] is the synergistic, and increasingly reciprocal, associations between age, motor competence, physical activity, perceived skill competence, health-related fitness, and weight status. The model of Stodden and colleagues [5] has since been examined to identify those health-enhancing paths most strongly supported by empirical evidence [6, 7]. In a narrative review, Robinson and colleagues [7] reported consistent evidence for a direct association between motor competence and physical activity, health-related fitness, and weight status. However, this was largely based on cross-sectional research.

Several systematic reviews and meta-analyses have provided additional support for these original paths, although these have often focused on a single path in the model of Stodden et al. [5, 8–11]. Notably, while the most recent review by Barnett and colleagues [6] supported the relationship of motor competence with fitness and weight status, the authors concluded there was insufficient evidence for the physical activity–motor competence path. This review mainly synthesized longitudinal and experimental evidence since 2015 (although cross-sectional evidence was also sought for the mediation mechanisms) and considered all analyses in each study, rather than only highlighting results in the hypothesized direction.

The original model of Stodden and colleagues [5], and Barnett et al.’s review [6] (which aimed to provide evidence on this model) focused on the relationship between motor competence and physical health. However, growing attention is being devoted to the centrality of motor competence in developing cognitive and social-emotional health domains (discussed further below) [12]. Subsequently, a recent commentary proposed expanding Stodden’s model [5] to encompass those paths associated with additional health outcomes, including metabolic health, mental health, cognition, and academic performance [13]. This was an important step, but a more nuanced and systematic view on mediators and moderators is still lacking. A review by Lubans et al. [14] provided broader insights to mental health outcomes associated with physical activity, including cognitive and emotional outcomes, but in this model, there was no consideration of the role of motor competence in this relationship. Therefore, there is a need for synthesized information regarding social-emotional outcomes in this context.

An emergent evidence base suggests motor competence may have an important role in the development of cognitive and social-emotional outcomes, similar to that proposed for physical health [15–17]. Cognition is an umbrella term that has been defined as the mental processes that contribute to perception, memory, intellect, and action [18]. Cognitive processes are central to how people think and resolve problems and life-span challenges. Children’s cognition develops in a uniform fashion over time, with virtually all children showing similar changes in the way they think and act [19]. Social-emotional health refers to social-behavioral and mental health outcomes, and includes competencies such as self-regulation, inter-personal skills, and externalizing behaviors [20]. Development of these competencies provides children with a strong foundation to adapt and succeed within school, correlating with academic self-efficacy and academic performance [21]. Social-emotional skills can be positively shaped through interventions, and their importance to outcomes across different domains and life stages is proposed to be greater than other commonly cited factors (e.g., socio-economic status [SES]) [22]. Cognition and social-emotional functioning have a dynamic interdependency and are positively influenced by physiological and behavioral factors [23]. The Robinson et al. narrative review [7] presented initial evidence of a positive association between motor competence and aspects of cognitive development, highlighting this area as an essential focus of future studies. Research on the linkage of motor competence to cognitive and social-emotional outcomes has since grown steeply, furthering our understanding of the role of motor competence for positive trajectories of holistic health development [24]. Several reviews have synthesized the relationship of motor competence and wider motor skills with specific aspects of cognitive development, and in presenting largely inconsistent evidence have highlighted the complexity of interpreting this relationship, including acknowledging the role of confounding factors [25–27]. For motor competence, there are several proposed mediators and moderators that explain and constrain the relationship with cognitive and social-emotional development, respectively [28].

Physical activity that has a strong perceptual-motor underpinning is considered to have a key role in the relationship between motor competence and cognitive and social-emotional outcomes [29, 30]. In this respect, the quality of the motor movement is seen as crucial and not solely the dose and intensity of movement. The realist review of Pesce et al. [28] built upon this notion in highlighting the role of ‘contextualized mechanisms’, which may be physical, cognitive, emotional, and social in nature. Moreover, Pesce and colleagues [28] addressed how these mechanisms may specifically influence the relationship between qualitatively different physical activity and broader cognitive and social-emotional outcomes. Even during infancy, interventions that facilitate early motor development by challenging movement flexibility and adaption show a coupling of action with foundational executive functions [31]. As children age, executive functions are proposed to become more distinct, developing from a single factor in infancy to diverse, but still correlated, constructs in adolescence [32]. As such, some evidence has shown that by adolescence, the relationship between motor competence and cognition is increasingly domain-specific, with specific movement skills and activity participation associated with individual cognitive domains [33].

**Fig. 1:**
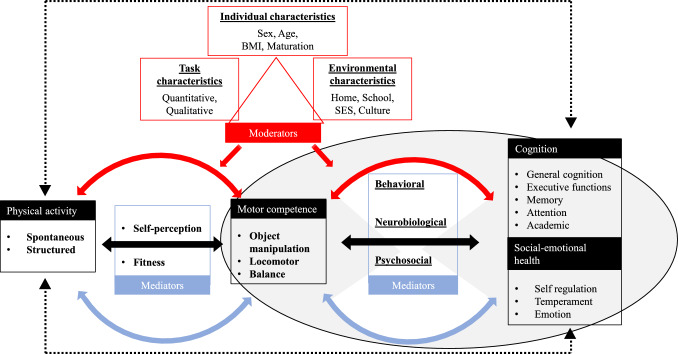
Conceptual model identifying the role of motor competence as a mediator between physical activity and domains of cognition and social-emotional health, with these causal pathways moderated by task, individual and environmental characteristics. *BMI* body mass index, *SES* socio-economic status

This review sought to present a conceptual model (Fig. 1) outlining the proposed influence of motor competence on developing cognitive and social-emotional outcomes during childhood and adolescence. The model provides a more comprehensive framework through which the position of motor competence can be evaluated, recognizing the dynamic interactions and associations underpinning its role.

### Conceptual Model

Our conceptual model (Fig. 1) builds on previous models that have focused on key aspects in isolation, such as the hypothesized moderated and mediated relationship of physical activity and mental health outcomes, and the direct and indirect relationship of motor competence with physical activity [1, 30]. Some of the hypothesized paths in these models have been extensively investigated; others need further research.

It is important to consider the theoretical rationales underpinning our broader model. Motor competence is positioned as a mediator between physical activity, cognition, and social-emotional health. Within the model, physical activity is the global term that comprises structured exercise or sport and spontaneous physical activity. For the purpose of the present review, we do not refer to any type of structured physical activity (i.e., exercise or sport), but specifically refer to physical activity tailored to prepare and support skill acquisition (‘deliberate preparation’, or ‘fundamental movement skill intervention’) [10, 34], whereas spontaneous physical activity is largely unstructured, freely chosen and characterized by exploration [35]. Both physical activity domains are proposed to have a crucial role in eliciting cognitive and social-emotional development, with free-play offering an autonomous child-directed context and structured practice providing a platform whereby children engage in cognitively challenging play [29, 36].

The model posits strong alignment and interaction between the underlying mechanisms of motor competence and cognitive development, particularly executive functions [37]. Consistent with the model by Lubans et al. [14], the proposed mechanisms that support the influence of motor competence on cognitive and social-emotional outcomes are set as neurobiological, psychosocial, and behavioral. However, the model does not pose such constraints, univocally linking individual mechanisms to specific outcomes (e.g., neurobiological mechanisms to cognitive outcomes and psychosocial/emotional mechanisms to wellbeing outcomes). Rather, it leaves the possibility open that, for instance, both neurobiological and psychosocial mechanisms may underlie physical activity effects on cognition in a differentiated and contextualized manner [28]. From a behavioral perspective, it is proposed that motor competence and cognitive processes are inextricably linked, with components of executive functions evident in the execution of gross motor skills [17, 38]. Children proficient in these skills will often engage in settings (e.g., sport practice and game-play) that are developmentally challenging from a motor and cognitive perspective and subsequently enhance motor and cognitive development [10]. The cognitive processes used to successfully control and adapt movement in these settings mirror those of strictly cognitive tasks [39].

The conceptual model, encompassing individual, task, and environmental constraints as potential moderators of the relationship between physical activity, motor competence, and cognitive/social-emotional outcomes, generates a cross-boundary intersection of Stodden’s model [5, 7] and Newell’s [40] insights on motor learning and development, through the triangulation of individual, task, and environmental constraints that influence motor development and learning. Although these theoretical approaches differ in their origins and goals, we make the case that we can capitalize on the ecological view of how individual, task, and environmental characteristics shape motor coordination to address how these characteristics, individually or jointly, may also moderate the association of motor competence with not only physical but also cognitive and social-emotional trajectories of health development.

The conceptual model proposes task characteristics as quantitative and qualitative. Along with the quantitative outcomes of physical activity (e.g., intensity), the qualitative characteristics are hypothesized to hold a fundamental role in moderating the association between motor competence and cognitive and social-emotional outcomes. Indeed, physical activity that is underpinned by decision-making, variability, and that is consistently challenging is seen to align with specific cognitive processes [41]. Therefore, whilst acknowledging the physiological changes in the brain induced by the quantitative characteristics of physical activity, such as the intensity, duration, or frequency, the qualitative pathways are similarly considered [31, 42]. Qualitative demands include behavioral factors, as well as cognitive, emotional, and social demands, such as characteristics relating to the quality of on-task engagement, interaction, and exploration [42, 43]. Indeed, within the school setting, low motor competence has been found to be associated with reduced on-task attention, and a withdrawal from those opportunities that promote motor development [44].

The conceptual model suggests key individual characteristics as moderators, including sex, weight status, age, and biological maturation. Biological maturation describes the progress towards a mature state, and involves processes occurring within bodily tissues, organs, and systems [45]. Increasing maturity is suggested to have sex-specific direct (kinematic) and indirect (psychological and behavioral) influences on aspects of motor competence [46–48]. Moreover, puberty-related hormonal changes contribute to a period of heightened social, emotional, and cognitive development, with specific cognitive functions coming ‘on-line’ at different stages [23]. Given that many children are entering adolescence with poor motor competence, more research is now being conducted on adolescent populations [49, 50]. However, continued reliance on chronological age to describe and group participants likely confounds the interpretation of reported associations and effects, and therefore fails to accurately consider the physiological, cognitive, and social development associated with maturation. The potential role of biological maturation and growth in the inter- and intra-individual variability in motor development, and the methods that can be adopted to capture the influence longitudinally, must be considered [51, 52]. With increasing age, weight status is correlated with motor competence and physical activity in children, with unhealthy weight status associated with less engagement in activities that promote the development of movement skills, an outcome occurring through direct (low competence) or indirect (self-perceived competence) mechanisms [5, 6]. Weight status is also hypothesized to have a bi-directional relationship with aspects of cognition (e.g., executive functions), with executive functions proposed to be important in managing obesity-related behaviors [53].

The conceptual model proposes environmental constraints such as the home, school setting, SES, and associated cultural factors. Within the home, parental social interactions, parental sensitivity, and involvement of parents (quantitatively and qualitatively) are all deemed influential on motor competence, physical activity, and cognitive development [54]. In addition, socio-economic status can further influence factors associated with the home (e.g., physical context, stimulation, lower parental expectation) along with promoting independent risk factors that include nutritional status and access to organized sport [55, 56]. Within the school, the pedagogical approaches that underpin sport and physical education (PE) delivery, the integration of physical activity across the curriculum, and the access to greenspace, may also play a moderating role on the highlighted pathways [57, 58].

In summary, the present review integratively focused on the developmental relationship of motor competence with cognitive and social-emotional outcomes. The aim was twofold: (i) to identify the potential mediating role of motor competence and related underlying mechanisms in the relationship between physical activity and its cognitive and social-emotional outcomes; and (ii) to identify the potential moderators in the interplay among physical activity, motor competence, and cognitive and social-emotional outcomes. Longitudinal evidence is focused on providing insight into cause and effect, and factors that constrain and differentiate the effects, such as individual and task characteristics, and factors that are still largely neglected [28].

## Methods

### Selection of Literature

This systematic review was registered (26/06/2020) with the International Prospective Register of Systematic Reviews (PROSPERO) and adhered to the Preferred Reporting Items for Systematic Reviews and Meta-Analyses (PRISMA) [59] statement for reporting systematic reviews and meta-analyses.

The review protocol can be accessed via https://www.crd.york.ac.uk/PROSPERO/#recordDetails. Five electronic databases (PubMed, Web of Science, Scopus, PsycINFO and SPORTDiscus) were searched (14/06/2020, and updated 11/06/2023) for peer-reviewed articles published only in English language, with no date restrictions applied. To formulate the search, search combinations were defined and implemented following discussion by all authors (Table 1).

**Table 1:** Search combinations used with each of the five electronic databases (PubMed, Web of Science, Scopus, PsycINFO and SPORTDiscus) to identify potential studies for inclusion

Variable	Search combination
Motor competence	‘motor skill*’ OR ‘movement skill*’ OR ‘motor development’ OR ‘gross motor’ OR ‘motor performan*’ OR ‘Motor Proficien*’ OR ‘motor abilit*’ OR ‘object manipulation’ OR ‘motor coordination’ OR ‘actual competen*’ OR ‘object control’ OR ‘locomotor skill*’ OR ‘motor proficiency’ OR ‘motor competen*’ OR ‘movement competenc*’ OR ‘motor fitness’ OR ‘fundamental movement’ OR ‘fundamental motor’ OR ‘basic movement’ OR ‘manipulative skill*’ OR ‘motor function*’ OR ‘athletic skill*’ OR ‘athletic competen*’ OR ‘skill proficiency’ OR ‘movement pattern’ OR ‘motor fitness’ OR ‘movement assessment’
Children	‘child*’ OR ‘adolescen*’ OR ‘student’ OR ‘teen*’ OR ‘youth’ OR ‘pediatric*’ OR ‘paediatric*’ OR ‘pube*’ OR ‘juvenil*’ OR ‘school*’ OR ‘youngster*’ OR ‘preschool*’ OR ‘kindergart*’ OR ‘kid’ OR ‘kids’ OR ‘playgroup*’ OR ‘play-group*’ OR ‘playschool*’ OR ‘prepube*’ OR ‘preadolescen*’ OR ‘junior high*’ OR ‘highschool*’ OR ‘senior high’ OR ‘young people*’ OR ‘young person’ OR ‘minors’
General cognition	‘cognit*’ OR ‘cognitive function’ OR ‘cognitive skill*’ OR ‘cognitive abil*’ OR ‘neurocognitiv*’ OR ‘cognitive development’ OR ‘neuro-cognitive’ OR ‘cognitive performance’ OR ‘cognitive control’
Cool executive functions	‘executive function*’ OR ‘problem solving’ OR ‘planning’ OR ‘reasoning’ OR ‘fluid intelligence’ OR ‘creativity’ OR ‘working memory’ OR ‘inhibition’
Hot executive functions	‘decision making’ OR ‘social cognit*’ OR ‘decision making’ OR ‘social cognition’ OR ‘emotional regulat*’ OR ‘cognitive flexibility’
Memory	‘operational memory’ OR ‘visuospatial memory’ OR ‘implicit memory’ OR ‘explicit memory ‘ OR ‘declarative memory’ OR ‘semantic memory’ OR ‘episodic memory’
Attention	‘selective attention’ OR ‘divided attention’ OR ‘sustained attention’ OR ‘vigilance’ OR ‘attention* orienting’, OR ‘focusing’ OR ‘executive attention’ OR ‘focus’
Academic	‘Academic achievement’ OR ‘academic performance’ OR ‘academic behavior’ OR ‘standardized testing’ OR ‘academic readiness’ OR ‘school readiness’ OR ‘task behavior’ OR ‘classroom behavior’
Social-emotional/self-regulation	‘self-regulat*’ OR ‘behavior self-regulat*’ OR ‘self-control’ OR ‘delayed gratification’ OR ‘temperamental control’ OR ‘emotion*’ OR ‘social’ OR ‘social skills’ OR ‘emotional skills’ OR ‘life skills’

*Word has been truncated to include different forms of the same word

### Eligibility Criteria

The eligibility for inclusion of studies was independently assessed by two authors (PH and MM) according to the following criteria:

(i) The review was constrained to studies targeting typically developing children and youth (aged 3–18 years). Therefore, studies of populations with known physical or cognitive impairment were not included.
(ii) Experimental and observational studies were required to have undertaken two or more assessment time points and measured, as a minimum inclusion criterion, motor competence and a cognitive and social-emotional development outcome at either time point.
(iii) Guided by the selection criteria presented by Barnett and colleagues [54], motor competence encompassed fundamental movement skills and motor coordination. Any study using a protocol that solely assessed wider aspects of ‘motor fitness’ or ‘physical fitness’ (i.e., strength, flexibility) was excluded. Similarly, any study that solely targeted fine motor skills was excluded. However, if motor competence and components of either motor/physical fitness or fine motor skills were analyzed and presented independently, the study was included. An exception was studies where motor competence and either motor/physical fitness or fine motor skills were analyzed as a composite score (e.g., McCarron Assessment of Neuromuscular Development [MAND]) [60].
(iv) Studies needed to assess a summary score of at least one aspect of motor competence (e.g., object manipulation and locomotor). Within a summary score, at least two skill assessments needed to be included (i.e., for object manipulation, overhand throw and kick).
(v) Studies that analyzed a single individual skill (e.g., overhand throw) were excluded.
(vi) The psychometric properties (i.e., construct and content validity) relating to specific process-oriented motor competence assessments were required to have been supported and presented in peer-reviewed evaluation and/or testing manuals. Also considered were any circuit-based approaches (e.g., Dragon Challenge [61] and Canadian Agility and Movement Skill Assessment [CAMSA]) [62].
(vii) Studies were included if the cognitive and social-emotional outcome(s) included a standardized test or a measure relating to any of the following: general cognition, executive functions, memory, attention, academic attainment/performance, and/or social-emotional development.
(viii) Studies were included if they reported statistical analyses of (potential) changes in cognitive function (general cognition, executive functions, memory, attention, academic) or indicators of social-emotional development (self-regulation, temperament, emotion) in relation to motor competence.
(ix) The review only included studies published in English in peer-reviewed journals, with no date restriction applied to the search.

All retrieved records were imported into the Rayyan systematic review platform for screening (Rayyan – Intelligent Systematic Review) [63]. Following the removal of duplicate studies, all authors were provided the opportunity to search their personal bibliographic libraries to identify additional articles for inclusion. Two authors (PH and MM) completed an initial assessment of eligibility on retrieved titles and abstracts independently. Following this, the same two authors completed a full-text screen of all potentially included articles. In instances where agreement on inclusion/exclusion could not be reached, three additional authors (LB, CP, NV) were consulted to review the articles, with each being discussed until a resolution was reached.

### Data Extraction and Reliability

Descriptive data for included studies were extracted and uploaded to an Excel document. Data extraction was completed by two authors (PH and MM) and verified by three further authors (LB, CP, and NV). For all studies, study characteristics (first author, year, sample size, study type, number of time points and study length, statistical procedure, mediating and/or moderating variables), participant characteristics (sex, age, country, biological maturity, weight status), motor competence assessment, cognitive and/or social-emotional assessment, and study results were imputed by a single author (PH). In addition, for experimental studies, the intervention content (high skill involvement, low skill involvement, or not available [28], and context (delivery type and setting) were coded. All extracted data were subsequently reviewed for accuracy (MM).

Risk-of-bias was assessed for individual studies by three authors (PH, MM, and NG). Prior to reviewing included studies, risk-of-bias was assessed on a subsample of five studies by the three authors (PH, MM, and NG) to ensure consistency, with any disagreements resolved in a consensus meeting with an additional author (PT). The same authors (PH, MM, and NG) then assessed the study quality of all studies, following the same process (Table 2). To assess study quality, the criteria established from reviewing the Strengthening the Reporting of Observation Studies in Epidemiology (STROBE) [64] statement were used. Following input from all authors, the criteria were amended to ensure appropriate applicability to the current review. This approach has been adopted in previous systematic reviews within this field [54, 65, 66]. The individual criteria were marked as ‘yes’ (a tick), ‘no’ (a cross), or ‘unclear’ (?).

**Table 2:** Methodological quality

Study	Study design	Study and assessment quality			Data analysis			
		1 Representative sampling	2 Minimal missing data	3 Valid motor competence assessment tool	4 Motor competence assessment reliabilities	5 Cognitive/social-emotional assessment validity	6 Appropriate statistical analysis	7 Covariates accounted for
Aadland et al. [71]	Observational	✓	✓	✓	x	✓	✓	✓
Aadland et al. [72]	Experimental	✓	✓	✓	x	✓	✓	✓
Battaglia et al. [73]	Experimental	x	?	?	x	?	✓	✓
Battaglia et al. [74]	Experimental	x	✓	✓	x	✓	✓	✓
Bedard et al. [75]	Experimental	x	✓	✓	x	✓	?	x
Berleze and Valentini [76]	Experimental	✓	✓	✓	✓	✓	✓	✓
Biino et al. [77]	Experimental	x	✓	✓	x	✓	✓	✓
Boat et al. [78]	Experimental	✓	✓	✓	x	✓	✓	x
Botha and Africa [79]	Experimental	✓	✓	✓	x	✓	?	x
Capio et al. [80]	Observational	x	✓	✓	x	✓	✓	✓
Chagas et al. [81]	Observational	?	✓	✓	x	✓	✓	✓
Condello et al. [82]	Experimental	✓	✓	✓	x	✓	✓	✓
De Oliveira et al. [83]	Experimental	x	x	✓	✓	✓	✓	✓
De Waal and Pienaar [84]	Observational	✓	x	x	x	x	✓	✓
Derman et al. [85]	Experimental	x	✓	✓	x	✓	✓	✓
Duncan et al. [86]	Experimental	✓	✓^a^	✓	✓	✓	✓	✓
Ericsson [87]	Experimental	x	?	✓	✓	✓	?	x
Fathirezaie et al. [88]	Experimental	x	?	✓	x	✓	✓	x
Gu et al. [89]	Observational	x	?	✓	✓	✓	✓	✓
Jaakkola et al. [90]	Observational	x	✓	✓	x	?	✓	✓
Jalilinasab et al. [91]	Experimental	✓	✓	✓	x	✓	✓	x
Katanić et al. [92]	Experimental	x	✓	✓	x	✓	✓	x
Koutsandréou et al. [93]	Experimental	✓	x	✓	x	✓	✓	x
Lee et al. [94]	Experimental	x	✓	✓	✓	✓	✓	x
Li et al. [95]	Experimental	✓	✓	✓	x	✓	✓	✓
Lin et al. [96]	Experimental	x	✓	✓	x	✓	✓	✓
Ludyga et al. [97]	Observational	x	?	✓	x	✓	✓	✓
MacDonald et al. [98]	Observational	x	✓	✓	✓	✓	✓	✓
Magistro et al. [99]	Experimental	x	✓	✓	✓	✓	✓	x
Minghetti et al. [100]	Experimental	x	✓	✓	x	✓	✓	✓
Mulvey et al. [101]	Experimental	✓	✓	✓	✓	✓	✓	✓
Niederer al. [102]	Observational	✓	✓	x	✓	✓	✓	✓
Nobre et al. [103]	Experimental	✓	x	✓	✓	✓	✓	✓
Oppici et al. [104]	Experimental	✓	✓	✓	x	✓	✓	✓
Osorio-Valencia et al. [105]	Observational	x	x	✓	x	✓	✓	✓
Pesce et al. [106]	Experimental	✓	x	✓	✓	✓	✓	✓
Riciardi et al. [107]	Observational	x	?	✓	x	✓	✓	✓
Rigoli et al. [108]	Observational	✓	✓	✓	x	✓	✓	✓
Robinson et al. [109]	Experimental	x	x	✓	✓	x	✓	x
Rodríguez-Negro et al. [110]	Experimental	x	x	✓	x	✓	✓	x
Rudd et al. [111]	Experimental	✓	✓	✓	x	✓	✓	✓
Son and Meisels [112]	Observational	✓	✓	✓	x	✓	✓	✓
Syväoja et al. [113]	Observational	x	✓	?	x	?	✓	✓
Taunton et al. [114]	Experimental	✓	?	✓	✓	✓	?	✓
Tocci et al. [115]	Experimental	✓	✓	✓	x	✓	✓	✓
Tseng et al. [116]	Experimental	x	✓	✓	✓	✓	✓	x
Vazou et al. [117]	Experimental	x	✓	✓	x	✓	✓	x
Zhang et al. [118]	Experimental	✓	✓	✓	x	✓	✓	x
Zysset et al. [119]	Observational	✓	✓	✓	x	✓	✓	✓
Totals by risk-of-bias criteria (49)		23	34	45	14	44	45	33

✓ met criteria, x did not meet criteria, ? unclear whether met criteria

^a^Criteria met for pre-post, but not met for 8 weeks post-intervention

### Criteria for Risk-of-Bias Assessment

The criteria for risk-of-bias assessment were as follows:

(i) Could the participant selection have introduced bias (i.e., were schools or students randomly selected or were other data provided to indicate population representativeness)? For experimental studies, was the process of randomization clearly outlined and adequately completed, including any between-group baseline differences?
(ii) Of those who consented to the study, did an adequate proportion have complete data for the outcome and all measures relating to this review (i.e., no more than 20% of data were missing from longitudinal studies ≤6 months, and no more than 30% for studies ≥6 months)?
(iii) Did the study report the sources and details of motor competence assessment? Were valid measures of motor competence used (validation in same age group published or validation data provided in the manuscript)?
(iv) Did the study report adequate reliability of motor competence assessment? For studies that used process-oriented motor competence assessments, adequate inter-rater reliability needed to be reported (i.e., intraclass correlation coefficient [or similar] ≥0.60) in addition to the above validity and reliability measures [67].
(v) Did the selected cognitive and social-emotional assessment provide evidence supporting construct validity (i.e., the extent to which the test provided a measure of the construct of interest)?
(vi) Did the study use appropriate statistical analyses for the study design?
(vii) Did the study report the sources and details for the assessment of potential correlates?

### Interpretation of Scientific Evidence

The effect size was estimated using the available data provided by the authors in each study (e.g., standardized regression coefficient or unstandardized beta, R^2^ for multiple regression, *F-*test, *T-*tests, means, standard deviations, and sample sizes) with two freely accessible effect size calculators (https://www.campbellcollaboration.org/escalc/html/EffectSizeCalculator-SMD22.php and https://www.danielsoper.com/statcalc/calculator.aspx?id=5). If authors reported correlation, Partial η^2^, and Cohen’s *d*, these were recorded as effect size. Conventional guidelines for the interpretation of the effect size were used [68–70].

The level of observational evidence for individual paths (e.g., object manipulation to working memory) was qualitatively synthesized using the approach favored by Barnett and colleagues [6]. For each path a percentage is presented, with this calculated from the number of significant analyses in the hypothesized direction, divided by the total number of analyses for that path. These percentages were then collated for each domain outcome (i.e., academic performance, working memory, and social behavior) to provide an indication of the level of evidence at a domain level. This collated influence was classified as either no association (0–33%), written as ‘0’; indeterminate/inconsistent (34–59%), written as ‘?’; or a positive ‘ + ’ or negative ‘ − ’ association (≥ 60%). When four or more studies found an association, it was classified as ‘ +  + ’ or ‘ −  − ’, accordingly. If there were fewer than three studies in the domain, the strength of evidence was considered insufficient to classify (I). To avoid a single study skewing the results, studies that included a large number of analyses (*N* ≥ 8) pertaining to a single path (i.e., object manipulation to working memory) were not included in the results synthesis.

Experimental evidence was also collated for individual paths (i.e., object manipulation to working memory). For each path, causal analyses of the relationship between specific motor and cognitive outcomes were prioritized and were synthesized using the same approach as used for observational evidence. In addition to the causal findings, studies where the analysis of outcomes was completed in parallel (e.g., the improvement of motor competence and cognitive outcomes analyzed and reported independently) and those studies that reported between-group differences of each outcome at post-intervention were also synthesized.

### Summary of Included Studies

**Fig. 2:**
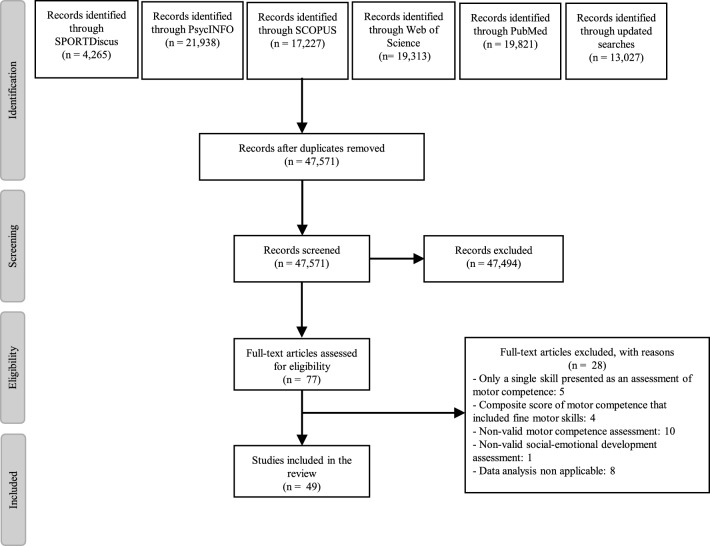
PRISMA flow diagram summarizing the literature review process

Following the removal of duplicates, the titles and abstracts of 47,571 studies were screened for eligibility (Fig. 2). Two authors (PH and MM) extracted descriptive data (Tables 3 and 4) for the 49 studies that met the inclusion criteria. Of the included studies, 15 used an observational study design [71, 80, 81, 84, 89, 90, 97, 98, 102, 105, 107, 108, 112, 113, 119] (Table 3), with 34 studies [72–79, 82, 83, 91–96, 99–101, 103, 104, 106, 109–111, 114–118] using an experimental design (Table 4).

**Table 3:** Descriptive information of included observational studies

Study	Country	Sample	Sex, *n*	Age (mean ± SD) at baseline	Motor competence assessment	Motor competence assessment method	Cognitive assessment	Social-emotional assessment	Study duration	Analysis	Study protocol
Aadland et al. (2017) [71]	Norway	1129	541 (g) 588 (b)	10.2 ± 0.3 y	MABC-2	Product-oriented Object manipulation	Numeracy, reading, English (NDET) inhibition (Stroop color and word test) cognitive flexibility (verbal fluency test, trail and making test) working memory (WISC-IV)		7 mo	SEM (mediation), Linear mixed model	Object manipulation **(T1)** to academic performance **(T2)** – through executive functions **(T2)**
Capio et al. (2022) [80]	Hong Kong	34	13 (g) 21 (b)	4.7 ± 0.3 y	TGMD-3	Process-oriented Locomotor skills, object manipulation	Verbal working memory (backward digit recall test), visuospatial working memory (Corsi block tapping test)		4 mo	Multivariate repeated measures ANCOVA	Locomotor skills, object manipulation **(T1)** to locomotor skills, object manipulation **(T2)** – through working memory
Chagas et al. (2022) [81]	Brazil	122	70 (g) 52 (b)	13.8 ± 0.7 y	KTK	Product-oriented Locomotor skills, dynamic balance	Academic attainment (Standardized regional tests)		5 mo	Univariate ANOVA	Locomotor skills, dynamic balance **(T1)** to academic attainment **(T2)**
de Waal and Pienaar (2020) [84]	South Africa	381	200 (g) 181 (b)	6.9 ± 0.4 y	BOT-2	Product-oriented Running speed and agility, strength, and balance	Numeracy, literacy, writing skills (the mastery of basic learning assessment), maths, home language, second language, natural sciences, and technology and life orientation (CAPS intermediate) language, maths (ANA), English, maths (NWPA)		7 y	Repeated measure ANOVA, SEM	Running speed and agility, strength, and balance **(T1)** to academic achievement **(T2)** Running speed and agility, strength, and balance **(T1)** to academic achievement **(T3)** Academic achievement **(T1)** to running speed and agility, strength, and balance **(T2)** Academic achievement **(T1)** to running speed and agility, strength, and balance **(T3)**
Gu et al. (2018) [89]	USA	141	69 (g) 72 (b)	5.4 ± 0.5 y	PE Metrics	Process-oriented Locomotor skills, object manipulation	Cognitive functioning (PedsQL™ Cognitive Functioning Scale)	Psychosocial function	1 y (academic)	Multiple regression, SEM	Locomotor skills **(T1)** to cognitive functioning scale, psychosocial function **(T2)** Object manipulation **(T1)** to cognitive functioning scale, psychosocial function **(T2)**
Jaakkola et al. (2015) [90]	Finland	325	162 (g) 162 (b)	13.1 ± 0.3 y	FMS Test Package	Product-oriented Leaping, shuttle-run, dribbling	Finnish language, mathematics, and history (academic grades)		34 mo	SEM (multi-group)	Leaping, shuttle-run, dribbling **(T1)** to academic performance **(T2)** Leaping, shuttle-run, dribbling **(T1)** to academic performance **(T3)** Leaping, shuttle-run, dribbling **(T2)** to academic performance **(T3)** Academic performance **(T1)** to leaping, shuttle-run, dribbling **(T2)** Academic performance **(T2)** to leaping, shuttle-run, dribbling **(T3)**
Ludyga et al. (2020) [97]	Germany	52	25 (g) 27 (b)	10.3 ± 0.5 y	MOBAK-5	Product-oriented Locomotor skills and object manipulation	Visual working memory (Sternberg task), event-related potentials		9 mo	SEM (path analysis)	Motor competence **(T1)** to reaction time **(T2)** Motor competence **(T1)** to iCNV amplitude **(T2)** Motor competence **(T1)** to Cue-P300 **(T2)**
MacDonald et al. (2016) [98]	USA	92	33 (g) 59 (b)	4.3 ± 0.7 y	PDMS-2	Process-oriented Object manipulation	Attentional flexibility, working memory, inhibitory control (HTKS)	Social behavior (SSIS-RS)	5 mo	SEM	Object manipulation **(T1)** to executive function **(T2)** Object manipulation **(T1)** to externalizing/hyperactivity **(T2)** Object manipulation **(T1)** to cooperation **(T2)** Object manipulation **(T1)** to self-control** (T2)**
Niederer et al. (2011) [102]	Switzerland	245	121 (g) 124 (b)	5.2 ± 0.6 y	Balance beam, Obstacle course	Circuit-based Agility, balance	Attention performance (KHV-VK), partial working memory performance (IDS)		9 mo	Mixed linear regression models	Agility **(T1)** to attention performance, partial working memory performance **(T2)** Balance **(T1)** to attention performance, partial working memory performance **(T2)**
Osorio-Valencia et al. (2018) [105]	Mexico	148	64 (g) 84 (b)	0–5 y	PDMS-2	Process-oriented Stationary balance, locomotor skills, object manipulation	McCarthy Scales of Children’s Abilities (verbal, quantitative, memory)		24 mo	Linear regression	Balance **(T1)** to cognitive abilities **(T2)** Locomotor skills **(T1)** to cognitive abilities **(T2)** Object manipulation **(T1)** to cognitive abilities **(T2)**
Ricciardi et al. (2021) [107]	USA	33,717		4–11 y	LAP-D	Product oriented Gross motor skills	LAP-D (cognitive, language), academic achievement (GPA, state standardized high-stakes test)	DECA (social-emotional skills)	7 y	OLS and binary logistic regression	School readiness: cognitive, language, social-emotional skills, motor competence **(T1)** to academic achievement **(T2)**
Rigoli et al. (2013) [108]	Australia	41	27 (g) 14 (b)	5–11 y	MAND	Product-oriented Gross motor skills	Visual working memory (The One-Back task)		18 mo	Multi-level mixed effects linear regressions	Gross motor skills **(T1)** to visual working memory **(T2)** Visual working memory **(T1)** to gross motor skills **(T2)**
Son and Meisels (2006) [112]	USA	12,583	6241 (g) 6342 (b)	49–83 mo (4.1–6.9 y)	ESI-R	Product-oriented Balancing, hopping, skipping, and walking backwards	Item response theory-based composite scores of reading and mathematics		15.8 mo and 21.5 mo	Hierarchical regression analyses	Gross motor skills **(T1)** to reading **(T2)** Gross motor skills **(T1)** to mathematics **(T2)**
Syväoja et al. (2019) [113]	Finland	954	496 (g) 458 (b)	12.5 ± 1.3 y	5-leaps test, throwing–catching combination test	Product-oriented Leaping, throwing-catching combination	Overall academic achievement (GPA)		2 mo	SEM, linear growth curve modelling	Leaping, throwing-catching combination **(T1)** to academic achievement **(T2)** Leaping, throwing-catching combination **(T2)** to academic achievement **(T3)** Academic achievement **(T1)** to leaping, throwing-catching combination** (T2)** Academic achievement **(T2)** to leaping, throwing-catching combination** (T3)**
Zysset et al. (2020) [119]	Switzerland	509	46.4% (g) 53.6% (b)	3.9 ± 0.6 y	ZNA 3–5	Process- and product-oriented Dynamic balance	Cognitive functioning (IDS-P)		12 mo	SEM	Motor competence **(T1)** to cognitive functioning **(T2)** Cognitive functioning **(T1)** to motor competence **(T2)**

*ANA* Annual National Assessments, *ANCOVA* Analysis of covariance, *BOT-2* Bruininks-Oseretsky Test of Motor Proficiency (2nd Edition), *ESI-R* Early Screening Inventory–Revised, *FMS* Fundamental Movement Skills, *CAPS* Curriculum and Assessment Policy, *DECA *Devereux Early Childhood Assessment, *g* girl, *GPA* Grade point average, *HTKS* Head Toes Knees Shoulders, *iCNV* initial contingent negative variation, *IDS-2* Intelligence and Developmental Scales 2nd Version, *IDS-P* Intelligence and Developmental Scales for Pre-School Children, *KHV-VK* Konzentrations-Handlungsverfahren für Vorschulkinder, *LAP-D* Learning Accomplishment Profile–Diagnostic*, LPA* latent profile analysis*, LVT* Leervoorwaarden Test, *KTK* Körperkoordinationstest für Kinder, *MABC* Movement Assessment Battery for Children*, MABC-2* Movement Assessment Battery for Children–2nd Edition, *MAND* McCarron Assessment of Neuromuscular Development, *mo* months, *MOBAK-5* Motoriche BasisKompetenzen-5, *NDET* Norwegian Directorate for Education and Training, *NWPA* North-West Provincial Assessment, *OLS* Ordinary Least Squares, *PDMS-2* Peabody Developmental Motor Scale (2nd Edition), *PE* physical education, *PedsQL*™ Pediatric Quality of Life Inventory, *R and A* Running speed and Agility, *SD* standard deviation, *SDQ* Strengths and Difficulties Questionnaire, *SEM* structural equation model, *SSIS-RS* Skills Improvement System–Rating Scales, *T* time point, *TCT-DP* Test for Creative Thinking–Drawing Production, *TGMD-3* Test of Gross Motor Development–Version 3, *WISC-IV* Wechsler Intelligence Scale for Children®–4th Edition, *y* years, *ZNA* Zurich Neuromotor Assessment

**Table 4:** Descriptive information of included experimental studies

Study	Country	Sample	Sex	Age (mean ± SD) at baseline	Intervention	Motor competence assessment	Motor competence assessment method	Cognitive assessment	Social-emotional assessment	Study protocol
Aadland et al. (2017) [72]	Norway	1129	541 (g) 588 (b)	10.2 ± 0.3 y	PA educational lessons (3 × 30-min per week), PA breaks during school lessons (5-min × 5 d/wk), PA homework (10-min × 5 d/wk). CG participated in curriculum-prescribed 90-min/wk of PE and 45-min/wk PA	MABC-2	Product-oriented Aiming, catching, shuttle run	Inhibition, cognitive flexibility, working memory (WISC-IV)		Experimental, 10 mo **T1:** Aiming, catching; inhibition, cognitive flexibility, working memory **T2:** Aiming, catching; inhibition, cognitive flexibility, working memory
Battaglia et al. (2018) [73]	Italy	119	51 (g) 68 (b)	CG: 4.3 ± 0.7 y IG: 4.8 ± 0.8 y	PE delivered 2 h/wk × 16 wk, including specific aims of developing body awareness, fundamental motor and perceptual-sensory skills. The CG participated in classroom activities for the same amount of time as the IG	TGMD	Process-oriented Object manipulation, locomotor skills	Pre-literacy skills (PRCR-2/2009)		Experimental, 16 w **T1:** Literacy readiness; object manipulation, locomotor skills **T2:** Literacy readiness; object manipulation, locomotor skills
Battaglia et al. (2019) [74]	Italy	1029	472 (g) 557 (b)	3–5 y	PE program delivered 2 h/wk × 16 wk, this included ludic-motor activities aimed at developing body awareness and fundamental motor and perceptual-sensory skills	TGMD (Italian Version)	Process-oriented Object manipulation, locomotor skills	Pre-literacy skills (PRCR-2/2009)		Experimental, 16 w **T1:** Literacy readiness; object manipulation, locomotor skills **T2:** Literacy readiness; object manipulation, locomotor skills
Bedard et al. (2018) [75]	Canada	11	5 (g) 6 (b)	45.6 ± 7.3 mo (3.8 ± 0.6 y)	The intervention was delivered for 1 h/wk × 10 wk. Each session consisted of 30-min of movement skill instruction, 15-min of free play, and a 15-min interactive reading circle	PDMS-2	Process-oriented Gross motor skills	Pre-literacy skills (PALS-PK)		Experimental, 10 w **T1:** Gross motor skills; pre-literacy skills **T2:** Gross motor skills; pre-literacy skills **T3:** Gross motor skills; pre-literacy skills **T4:** Gross motor skills; pre-literacy skills
Berleze and Valentini (2022) [76]	Brazil	100	50 (g) 50 (b)	7.0 ± 0.7 y	Mastery Climate Group participated in a high autonomy and child-centered approach with nutritional orientation. 28 wk (2 sessions/wk, with each session 90-min, of these, 41 sessions with a focus on motor competence; 15 with a focus on health) Parents involved in 6 sessions Control group participated in a low autonomy and teacher centered approach. PA and motor skill lessons 28 wk (2 sessions/wk, with each session 90-min)	TGMD-2	Process-oriented Object manipulation, locomotor skills		Social Acceptance (Pictorial scale of perceived competence and social acceptance), Engagement	Experimental, 28 w **T1:** (Pre-intervention eligibility) Object manipulation, locomotor skills **T2:** PSPSCA: Self-perceptions – cognitive, motor, social, global self-worth. Daily routine: screen time, play time, activities at home and transportation **T3**: Object manipulation, locomotor skills; PSPCSA and daily routine
Biino et al. (2021) [77]	Italy	36	19 (g) 7 (b)	60.6 ± 7.4 mo (5.1 ± 0.6 y)	IG performed the respective PA interventions, composed of 45-min sessions twice a wk IG (1) attended a PA course enriched with cognitive demands tailored to challenge core EFs IG (2) attended a swimming course of the same frequency and duration. CG did not attend any structured PA additional to normal daily activities	PDMS-2	Process-oriented Gross motor skills	Executive functions (Forward Word Span Test, Trail Making Test)		Experimental, 12 w **T1:** Motor competence; executive functions **T2:** Motor competence; executive functions
Boat et al. (2022) [78]	Italy	192	102 (g) 90 (b)	CG: 8.5 ± 0.8 y IG: 8.5 ± 0.9 y	The 16-wk intervention involved a number of games within the physically active lessons, each linked to a specific mathematical or English language component. Each lesson contained a warm-up, an explanation, two main activities, and a summary	TGMD-3	Process-oriented Object manipulation, locomotor skills	Cognitive function (WISC-IV)		Experimental, 12 w **T1:** Motor competence; cognitive function **T2:** Motor competence; cognitive function
Botha and Africa (2020) [79]	South Africa	97		6–7 y	Intervention delivery focused primarily on perceptual-motor skills and incorporated different letters and shapes into gross motor activities. The IG participated in the intervention twice a week for 60-min	BOT-2	Product-oriented Upper limb coordination, balance, running speed and agility	Letter knowledge (ESSI reading and spelling tests)		Experimental, 12 w **T1:** Upper limb coordination, balance, running speed and agility; letter knowledge **T2:** Upper limb coordination, balance, running speed and agility; letter knowledge
Condello et al. (2021) [82]	Italy	181	90 (g) 91 (b)	10–11 y	The intervention occurred across 6 mo during 1 h/wk PE class. IG received an enriched multisport PE intervention focusing on life skills, and challenging EF. The CG received the traditional PE curriculum	AST	Circuit-based approach Locomotor skills, manipulative and stability	Executive functions (RNG, GPAI)	Prosocial and antisocial behavior (MASCS)	Experimental, 6 mo **T1:** Motor competence, executive functions, prosocial and antisocial behavior **T2:** Motor competence, executive functions, prosocial and antisocial behavior
De Oliveira et al. (2018) [83]	Australia	511	254 (g) 257 (b)	5.4 ± 3.6 y	Animal Fun (AF) implemented for 30-min/d × 4 d/wk for a minimum of 10 wk. AF focuses on embedding gross and fine motor development and social-emotional development into the learning curriculum. CG classes followed normal curriculum	BOT-2 SF, MABC-2	Product-oriented Aiming, catching and balance	Intellectual functioning (WPPSI-III)		Experimental, 18 mo **T1**: Aiming, catching and balance, intellectual functioning **T2:** Aiming, catching and balanc**e** **T3:** Aiming, catching and balance
Derman et al. (2020) [85]	Turkey	45	20 (g) 25 (b)	54.0 ± 3.4 mo (4.5 ± 0.3 y)	A quasi-experimental quantitative design including a pre-test–post-test and experimental–control group was used to assess the effect of play-based math activities on different developmental areas (personal-social, fine motor, language and gross motor development). The IG performed 14 play-based math activities for 1 h × 2 d/wk/8 wk The CG continued their education within the framework of the Ministry of National Education-Preschool Education Curriculum	Denver II Developmental Screening Test	Product-oriented	Language (Denver II Developmental Screening Test)	Personal–social (Denver II Developmental Screening Test)	Experimental, 2 mo **T1:** Personal social, language, gross motor skills **T2:** Personal social, language, gross motor skills
Duncan et al. (2019) [86]	UK	74	35 (g) 39 (b)	3–4 y	Combined movement and story-telling IG received across 6 wk, 2 × 20–30-min sessions received twice/wk. Two CGs: one received story-telling only, the second CG received movement in isolation	TGMD-2	Process-oriented Run, jump, catch and overarm throw	Language ability (BAS3)		Experimental, 6 wk **T1:** Motor competence, language ability **T2:** Motor competence, language ability **T3:** Motor competence, language ability
Ericsson (2008) [87]	Sweden	251		School year 1–3	The IG PE extended from 2 to 3 lessons and different local sports clubs had PA for 2 lessons/wk. IG had PA for 5 lessons/wk and if needed, 1 extra lesson of motor training per week. The CG had only the school’s routine PE for 2 lessons/wk	MUGI Observation	Process-oriented Balance, bilateral coordination, hand–eye coordination	Conners’ questionnaire (teachers’ and parents’ conceptions of children’s attention ability and impulse control) academic performance		Experimental, 3 y **SY1:** Motor observations, reading development, teachers’ and parents’ conceptions of children’s attention ability and impulse control **SY2:** Motor observations, academic performance, teachers’ conceptions of children’s attention ability and impulse control **SY3:** Motor observations, word and reading test, parents’ conceptions of children’s attention ability and impulse control
Fathirezaie et al. (2021) [88]	Iran	30	17 (g) 13 (b)	5.5–6.5 y	Intervention conducted over 12 sessions (4-h sessions). The intervention for the outdoor physical activity group included nature play. The second group of the study consisted of typical kindergartens who performed their activities in the indoor space of the kindergarten	BOT	Product-oriented Running speed and agility, balance		Social maturation (VSMS)	Experimental, 12 wk **T1:** Running speed and agility, balance, social maturation **T2:** Running speed and agility, balance, social maturation
Jalilinasab et al. (2021) [91]	Iran	84	42 (g) 42 (b)	9.6 ± 1.1 y	The IG received Brain Gym training for 8 wk × 2 sessions/wk (45-min session), Brain Gym is a program aimed at improving motor, cognitive, and social learning. The CG continued with routine lives and learning	TGMD-3	Process-oriented Locomotor skills and object manipulation		Social skills (MESSY)	Experimental, 8 wk **T1**: Locomotor skills, ball skills, social skills **T2:**: Locomotor skills, ball skills, social skills **T3:** Locomotor skills, ball skills, social skills
Katanić et al. (2021) [92]	Serbia	47	24 (g) 23 (b)	IG: 6.4 ± 0.3 y CG: 5.9 ± 0.3 y	The IG received aerobic training for children over a period of 12 wk × 3 training sessions/wk (30 min each)	BOT-2	Product-oriented Balance	Cognitive development, cognitive maturity (School maturity test)		Experimental, 12 wk **T1:** TZŠ + cognitive maturity test, balance **T2**: TZŠ + cognitive maturity test, balance
Koutsandréou et al. (2016) [93]	Germany	71	39 (g) 32 (b)	9.4 ± 0.6 y	Children randomly assigned to a cardiovascular exercise, a motor exercise, or a control group. Intervention period that involved 10 wk of an additional after-school exercise regimen, which took place 3 × per wk for 45 min	HGMT	Circuit-based Balance, rhythm, spatiotemporal orientation, and motor adaption to moving objects	Working memory processing (The Letter Digit Span)		Experimental, 10 wk **T1:** Balance, rhythm, spatiotemporal orientation, and motor adaption to moving objects, working memory processing **T2:** Balance, rhythm, spatiotemporal orientation, and motor adaption to moving objects, working memory processing
Lee et al. (2020) [94]	USA	31	19 (g) 12 (b)	6.7 ± 1.0 y	An 8-wk FMS intervention, embedded in an afterschool program 3 × per wk (60 min each time) in 24 sessions, CG followed a regular afterschool program (e.g., unstructured child free-play, drawing, reading, and/or academic tutoring)	TGMD-2	Process-oriented Locomotor skills and object manipulation	Cognitive functioning (PedsQL™ (Cognitive Functioning Scale)		Experimental, 8 wk **T1:** Locomotor skills and object manipulation, cognitive functioning scale **T2:** Locomotor skills and object manipulation, cognitive functioning scale
Li et al. (2022) [95]	Hong Kong	79	48 (g) 31 (b)	S + M: 9.7 ± 0.7 y M: 9.6 ± 0.6 y CG: 9.6 ± 0.6 y	Participants were assigned to either a blended intervention (S + M) group, a single PA break group (M), and a CG. The (S + M) group used sit-stand desks for at least 1 h/d on average across the wk. PA breaks were up to 15 min in duration and 2 × per day across the week. (M) children participated in a PA recess during recess time. CG adhered to their regular class schedules and lesson delivery format	CAMSA	Circuit-based approach	Inhibitory control (Eriksen flanker task)		Experimental, 13 wk **T1:** Motor competence, inhibitory control **T2:** Motor competence, inhibitory control **T3:** Motor competence, inhibitory control
Lin et al. (2021) [96]	Taipei	52	26 (g) 24 (b)	IG: 8.5 ± 1.1 y; CG: 8.7 ± 1.1 y	8-wk motor skills-based PA program (gymnastics), 2 sessions/wk (90 min) specifically focusing on interlimb gross motor movements. CG instructed to maintain routine activities	MABC-2	Product-oriented Aiming, catching and balance	Working memory (Delayed-matching working memory task), EEG recording		Experimental, 8 wk **T1:** Aiming, catching and balance; working memory, EEC recording **T2:** Aiming, catching and balance; working memory, EEC recording
Magistro et al. (2022) [99]	Italy	82	37 (g) 45 (b)	IG: 6.6 ± 0.3 y; CG: 6.6 ± 0.3 y	The intervention consisted of integrating physical activity bouts in all mathematics teaching hours (8 h/wk), implemented for 2 school years. It consisted of 75 different games, each with 4 possible variations. Each game was connected to a specific mathematical element. The control condition consisted of continuing the usual mathematics teaching program	TGMD-3	Process-oriented Locomotor skills and object manipulation	Cognitive function (BVN 5–11 battery)		Experimental, 2 y **T1:** Locomotor skills and object manipulation; cognitive function **T2:** Locomotor skills and object manipulation; cognitive function **T3:** Locomotor skills and object manipulation; cognitive function **T4:** Locomotor skills and object manipulation; cognitive function
Minghetti et al. (2021) [100]	Switzerland	68	23 (g) 23 (b)	4.9 ± 0.7 y	Children (and senior participants) assigned intergenerational, peer or a CG. 25 weekly exercise sessions lasting 45 min each. Dynamic balance exercises (walking forwards, backwards, sideways, over objects such as ropes or unstable surfaces) as well as object control skills such as throwing, aiming, rolling and catching a variety of objects	TGMD-2	Process-oriented Locomotor skills and object manipulation		Social-emotional skills (KOMPIK)	Experimental, 25 wk **T1:** Locomotor skills and object manipulation, social skills (self-assertion and cooperation), emotional skills (empathy and emotional regulation), wellbeing and social relationships **T2:** Locomotor skills and object manipulation, social skills (self-assertion and cooperation), emotional skills (empathy and emotional regulation), wellbeing and social relationships
Mulvey et al. (2018) [101]	USA	107	58 (g) 49 (b)	5.4 ± 0.8 y	Intervention condition participated in the SKIP motor skill intervention twice weekly over 6 wk for 30 min. Children in the control condition participated in the center’s ‘business as usual’ condition 5 d/wk for 30 min	TGMD-2	Process-oriented Locomotor skills and object manipulation	Behavioral regulation (HTKS task)		Experimental, 6 wk **T1:** Locomotor skills and object manipulation; behavioral regulation **T2:** Locomotor skills and object manipulation Behavioral regulation
Nobre et al. (2022) [103]	Brazil	280	140 (g) 140 (b)	7–10 y	A 12-wk (3 × per wk/36 lessons/ 140-min session) intervention was designed following the mastery climate guidelines. 5–7 stations were implemented, containing a diverse range of body and space awareness, balance, locomotor skills, and ball skills tasks. The CG participated in a program that provided recreational sports, arts, academic reinforcement, and crafts that follow the scholar curriculum	TGMD-2	Process-oriented Locomotor skills and object manipulation	Academic attainment (The School Performance Test)		Experimental, 12 wk **T1:** Locomotor skills and object manipulation; academic attainment **T2:** Locomotor skills and object manipulation; academic attainment
Oppici et al. (2020) [104]	Australia	80	48 (g) 32 (b)	8.8 ± 0.7 y	IG undertook practical dance choreography 2 × per wk for 60 min × 7 wk (14 lessons). CG undertook standard school PE curriculum classes	CAMSA	Circuit-based approach	Working memory and other cognitive functions (NIH Toolbox)		Experimental, 7 wk **T1:** Working memory capacity, motor competence **T2:** Working memory capacity, motor competence
Pesce et al. (2016) [106]	Italy	460	230 (g) 230 (b)	5–10 y	The two experimental interventions differed from one another in that in one the PA games were altered to involve a higher amount of mental engagement and challenge executive functions (cognitively engaging specialist-led intervention). All children participated in PE for 1 h/wk, and intervention duration was 6 mo	M-ABC	Product-oriented Object manipulation, static and dynamic balance	Inhibition and working memory updating (RNG task), attention (CAS)		Experimental, 6 mo **T1:** Object manipulation, static and dynamic balance, inhibition and working memory updating (RNG task), attention (CAS) **T2:** Object manipulation, static and dynamic balance, inhibition and working memory updating (RNG task), attention (CAS)
Robinson et al. (2016) [109]	USA	113	57 (g) 56 (b)	51.9 ± 6.5 mo (4.3 ± 0.5 y)	Children randomly assigned to a CHAMP treatment or control. Children in the CHAMP group replaced their outdoor recess with CHAMP 3 d/wk for 5 wk (15 × 40-min sessions). The control condition was the typical movement program. CHAMP looks to enhance motor skills, perceived physical competence, and PA	TGMD-2	Process-oriented Locomotor skill and object manipulation		Self-regulation (The delay of gratification snack task of the Preschool Self-Regulation Assessment)	Experimental, 5 wk **T1**: Locomotor skills and object manipulation, self-regulation **T2:** Locomotor skills and object manipulation, self-regulation
Rodríguez-Negro et al. (2020) [110]	Spain	249		9.6 ± 1.2 y	Intervention held during PE lessons for 8 wk (sessions of 90-min). The intervention included 3 programs; balance intervention program, game-based program, and drama learning program with the program effects on school-age children’s cognitive (creativity, attention and impulse control) and motor competence measured	MABC-2	Product-oriented Object manipulation, static and dynamic balance	Creativity, attention and impulse control (CARAS-R test, CREA test (creativity)		Experimental, 8 wk **T1**: Creativity, attention, impulse control; static balance, dynamic balance, aiming and catching **T2:** Creativity, attention, impulse control; static balance, dynamic balance, aiming and catching **T3:** Creativity, attention, impulse control; static balance, dynamic balance, aiming and catching
Rudd et al. (2021) [111]	Australia	62	29 (g) 33 (b)	6.6 ± 0.5 y	8-wk dance curriculum intervention. 8-wk control period first prior to 8-wk intervention. PE classes were 16 sessions of 50-min each. Two IG were randomly assigned following the control period (choreography and dance group)	CAMSA	Circuit-based approach	Executive functions (NIH Toolbox), working memory capacity (List sorting working memory test), cognitive flexibility (DCCS test), Inhibitory control (The Flanker test)		Experimental, 8 wk **T1:** Working memory capacity, cognitive flexibility, inhibitory control, motor competence **T2:** Working memory capacity, cognitive flexibility, inhibitory control, motor competence **T3:** Working memory capacity, cognitive flexibility, inhibitory control, motor competence
Taunton et al. (2018) [114]	USA	80	39 (g) 41 (b)	55.4 ± 7.0 mo (4.6 ± 0.6 y)	Children in the experimental condition participated in the SKIP motor skill intervention twice weekly for 6 wk for 30 min during each session (360 min), and they participated in ‘business as usual’ (i.e., regularly implemented recess) the other 3 d/wk throughout the study	TGMD-2	Process-oriented Locomotor skills and object manipulation		Surgency, negative affect, and effortful control (CBQ)	Experimental, 6 wk **T1:** Locomotor skills and object manipulation **T2**: Surgency, negative affect, and effortful control **T3**: Locomotor skills and object manipulation
Tocci et al. (2022) [115]	Italy	95	48 (g) 47 (b)	7.8 ± 1.3 y	The intervention was performed during PE for 1 h once a week, lasting 6 mo and a total amount of 24 intervention hours. The intervention was designed in a theory-based manner, using a constraints-led and cognitive stimulation approach. To foster the deliberate, cognitively engaging mode of creativity, teachers also manipulated the time constraints on the search for solutions. Teachers of the CG were instructed to perform their ‘business as usual’	MABC-2	Product-oriented Object manipulation, static and dynamic balance	Executive functions (the Random Number Generation task), Creative thinking (Torrance Test of Creative Thinking)		Experimental, 12 wk T1: Executive functions; creative thinking; object manipulation, static and dynamic balance T2: Executive functions; creative thinking; object manipulation, static and dynamic balance
Tseng et al. (2022) [116]	Taiwan	10	1 (g) 9 (b)	10.5 ± 0.7 y	Participants participated in a PA program twice a week after school for 12 wk. It was held twice a week for 90 min, and each session consisted of 5 min of warmup, 20 min of fitness skills practice, 40 min of fundamental skills practice, 20 min of game set activity, and 5 min of cool down	MABC-2	Product-oriented Static and dynamic balance	Executive functions (Modified task-switching paradigm)		Experimental, 12 wk T1: Executive functions, object manipulation, static and dynamic balance T2: Executive function, object manipulation, static and dynamic balance
Vazou et al. (2020) [117]	USA	39	18 (g) 21 (b)	7.7 ± 1.5 y	Children ages 6–11 y were enrolled in one of two programs: a rhythmic program (active learning of rhythmic gross motor actions to different songs) and a generalized PE program (developmentally appropriate active learning for gross motor actions), both meeting for 30-min × 2 sessions/wk, for 7 wk	MABC-2	Product-oriented Static and dynamic balance	Non-verbal intelligence (KBIT-2), attention and behavioral control (SWAN rating scale), executive functions (Flanker Fish test)	Motivational climate (Peer Motivational Climate in Youth Sport Questionnaire)	Experimental, 7 wk **T1**: Balance, cool and hot EF and social-emotional factors **T2:** Balance, cool and hot EF and social-emotional factors
Zhang et al. (2022) [118]	China	109	55 (g) 54 (b)	IG: 4.5 ± 0.3 y; CG: 4.5 ± 0.3 y	The intervention group received physical activity interventions (3 × 40-min sessions weekly), while children in the control group engaged in regular activities. Activities included two types of games. Type 1 games focused on motor learning with the purpose of allowing children to acquire fundamental movement skills. Type 2 games were based on type 1 games but incorporated more cognitive rules that were specifically designed to foster children's cognitive abilities	MABC-2	Product-oriented Object manipulation, static and dynamic balance	Working memory (1-back task)		Experimental, 12 wk T1: Working memory, object manipulation, static and dynamic balance T2: Working memory, object manipulation, static and dynamic balance

*AST* Athletic skills track,* b* boys*, BAS3* British Ability Scales–3, *BOT-2* Bruininks-Oseretsky Test of Motor Proficiency (2nd Edition), *BOT-2 SF* Bruininks-Oseretsky Test of Motor Proficiency (2nd Edition) short form, *CAMSA* Canadian Agility and Movement Skill Assessment, *CARAS-R* Test of Perception of Differences–Revised, *CAS* cognitive assessment system, *CBQ* Children’s behavior questionnaire*, CG* control group*, CHAMP* Children’s Health Activity Motor Program*, CREA test* Creative Intelligence Test, *DCCS* Dimensional change card sort, *EEG* Electroencephalogram*, EF* executive functions*, GPAI* Game Performance Assessment Instrument*, g* girls, *h* hours, *HGMT* Heidelberg Gross-Motor Test, *HTKS* Head Toes Knees Shoulders, *IG* intervention group*, KBIT-2* The Kaufman Brief Intelligence Test, 2nd Edition, *M* Move group, *MABC* Movement Assessment Battery for Children, *MABC-2* Movement Assessment Battery for Children–2nd Edition, *MASCS* Multi-Source Assessment of Children's Social Competence, *MESSY* Matson Evaluation of Social Skills with Youngsters, *min* minutes, *mo* months, *MUGI* Motorisk Utveckling som Grund för Inlärning Observation instrument, *NIH Toolbox* National Institutes of Health Toolbox, *PA* physical activity, *PALS-PK* Phonological Awareness Literacy Screening: preschool, *PE* physical education, *PDMS-2* Peabody Developmental Motor Scale (2nd Edition), *PedsQL*™ Pediatric Quality of Life Inventory, *PSPCSA* The Pictorial Scale of Perceived Competence and Social Acceptance*, RNG* random number generation, *S* + *M* Stand + Move group, *SKIP* Successful Kinesthetic Instruction for Preschoolers, *SWAN* Strengths and Weaknesses of Attention-Deficit/Hyperactivity Disorder Symptoms and Normal Behavior Scale, *SY* school year, *TGMD* Test of Gross Motor Development*, TGMD-2* Test of Gross Motor Development–Version 2, *TZŠ* + Test zrelosti za skolu,* T* time point, *VSMS* Vineland Social Maturity Scale, *w* weeks, *WISC-IV* Wechsler Intelligence Scale for Children®–4th Edition, *WPPSI-III* Wechsler Preschool and Primary Scale of Intelligence–Version 3,* y* years

The majority of included studies were conducted in the USA [89, 94, 98, 101, 107, 109, 111, 113, 117] and Italy [73, 75, 77, 79, 82, 99, 106, 115], with four studies conducted in Australia [83, 104, 108, 111], and three completed in Switzerland [100, 102, 119] and Brazil [76, 81, 103]. A further two studies were conducted in each of the following countries: South Africa [79, 84], Finland [90, 113], Hong Kong [80, 95], Iran [88, 91], Norway [71, 72], and Germany [93, 97]. In addition, a single study was conducted in Canada [75], China [118], Mexico [105], Serbia [92], Spain [110], Sweden [87], Taipei [96], Taiwan [116], Turkey [85], and the UK [86].

Preschool-aged children (3–5 years) were recruited to participate in 21 studies [73–75, 77, 79, 80, 83, 85, 86, 89, 92, 98, 100, 101, 105, 107, 109, 112, 114, 118, 119], pre-adolescent children (6–9 years) in 20 studies [76, 78, 84, 87, 88, 91, 93–96, 99, 102–104, 106, 108, 110, 111, 115, 117], and only eight studies recruited adolescent participants (10–18 years) at baseline [71, 72, 81, 82, 90, 97, 113, 116]. Although the studies included within this review were characterized by a wide range of sample sizes (10–33,717 children), 51% of included studies had sample sizes ≥ 100 participants.

For all included studies, a high rate of agreement (88%) was observed between researchers (PH, MM, and NG) on the risk-of-bias assessment (Table 2). In instances where initial agreement was not reached on individual criteria, the study was further reviewed, and a final decision agreed upon with an additional author (PT). Only 47% of included studies were found to have achieved representative sampling and only 69% of studies presented an adequate level of data completion for participants. Although the majority of studies included validation data for the motor competence assessment (validation in same age group published or validation data provided in the manuscript) and assessment of cognitive and social-emotional development, only 29% of studies reported adequate reliability for the motor competence assessment used in the current study. When assessing the data analysis of included studies, over 90% of studies were found to use an appropriate approach to data analysis, with 67% of included studies considering covariates. Statistical mediation of physical activity effects on cognitive and social-emotional outcomes by motor competence was only included in < 5% of studies. Similarly, the role of task, individual, and environmental characteristics in moderating the association between motor and cognitive and social-emotional outcomes was only explicitly analyzed in 29% of studies.

#### Motor Competence Assessment

For studies that met the inclusion criteria, motor competence was assessed using process-oriented, product-oriented, and circuit-based instruments. A process-oriented assessment was used in 21 studies [73–78, 80, 86, 87, 89, 91, 94, 98–101, 103, 105, 109, 114, 119], a product-oriented assessment in 22 studies [71, 72, 79, 81, 83–85, 88, 90, 92, 96, 97, 106–108, 110, 112, 113, 115–117], with the remaining six studies [82, 93, 95, 102, 104, 111] using a circuit-based approach to assessment. Collectively, versions of the Test of Gross Motor Development (TGMD) [120] were the most selected process-oriented assessments, with four studies using the Peabody Developmental Scales, 2nd Edition (PDMS-2) [121], and further studies using the Motorisk Utveckling som Grund för Inlärning (MUGI) Observation instrument [122] and the PE and Metrics assessment [123]. Several product-oriented instruments were used, with the Bruininks-Oseretsky Test of Motor Proficiency, 2nd Edition (BOT-2) [124] and Movement Assessment Battery for Children, 2nd Edition (MABC-2) [125] present in multiple studies. For the remaining studies, the Denver II Developmental Screening Test [126], fundamental movement skills (FMS) Test Package [127], Körperkoordinationstest für Kinder (KTK) [128], the Learning Accomplishment Profile-Diagnostic (LAP-D) [129], Movement Assessment Battery for Children (M-ABC) [130], MAND [60], Motoriche BasisKompetenzen (MOBAK-5) [131], 5-leaps test and throwing-catching combination test [132], Zurich Neuromotor Assessment (ZNA 3–5) [133], and the Early Screening Inventory-Revised (ESI-R) [134] were all used in single studies. The six circuit-based approaches to assessment were the Athletic Skills Track (AST) [135], CAMSA [62], Heidelberg Gross Motor Test [136], and the Balance beam and Obstacle course assessment [137]. In 22 studies [73–77, 81, 82, 85–87, 89, 91, 93, 94, 97, 100, 101, 104, 107–109, 111], a composite-level outcome of motor competence was analyzed, with the remaining studies assessing object manipulation skills, locomotor skills, and balance competence.

#### Cognitive and Social-Emotional Assessment

Cognitive and social-emotional assessment validity was deemed acceptable for 44 (90%) of the included studies within the current review. Twelve studies investigated the relationship between motor competence and social-emotional development aspects [76, 82, 85, 88, 89, 91, 98, 100, 107, 109, 114, 117]. In contrast, 43 studies [71–75, 77–87, 89, 90, 92–99, 101–108, 110–113, 115–119] included an analysis of the relationship between motor competence and cognitive functioning aspects. In the 21 studies that included children of pre-school age, domains of executive functioning, pre-literacy score, intellectual functioning, and social-emotional development were the assessed outcomes. The studies that included pre-adolescent children assessed the relationship between motor competence and cognitive development domains (i.e., executive functions), academic performance, and social-emotional development. The eight studies that comprised adolescent samples [71, 72, 81, 82, 90, 97, 113, 116] included aspects of cognitive development (i.e., working memory, creativity, attention, and impulse control) and academic performance as their assessed outcomes.

#### Exposure Characteristics

Of the 15 observational studies, the length of study ranged between 2 months and 7 years, with 10 of the studies including two time points and the remaining studies all having three measurement time points. The 34 experimental design studies had a study length of between 5 weeks and 3 years. The intervention delivery included individual, environmental, and physical activity characteristics. Interventions primarily occurred as part of the school day and included the promotion of motor competence within an enriched and developmentally appropriate PE context.

## Results

### Observational Evidence

#### Motor Competence and Cognition

Seven observational studies (Table 5) assessed the longitudinal association of motor competence and aspects of academic performance, (pre)literacy, and intellectual functioning [71, 81, 84, 90, 107, 112, 113]. Of these studies, two presented some supportive evidence for the relationship of composite-level motor competence (6/21 analyses) and locomotor skills (17/54 analyses) with academic performance in adolescents [90, 113]. A similar level of evidence was found for the reverse path (academic performance–motor competence), with two studies finding a positive relationship of academic performance with leap skill (5/8 analyses) and composite motor competence (1/2 analyses) [90, 113]. At pre-school and pre-adolescent ages, the evidence was less supportive, with composite-level motor competence, locomotor skills, and balance found to have a negligible [84, 112] or negative [107] relationship with academic performance, when adjusted for individual confounders (i.e., sex, age, body mass index [BMI]). Furthermore, across all studies, a consistent construct-specific and/or academic subject-specific relationship was not found.

**Table 5:** Analyses and results (observational studies)

Motor competence and cognitive and social-emotional outcomes	Significant improvement (Reported effect sizes)	No significant improvement (Reported effect sizes)	Summary of results (Analyses reporting a significant improvement/ total analyses)
**Studies classified by cognitive and social-emotional outcome**			
	**Academic performance**		(O)
Catching		Aadland et al. [71] *SES* (3/3)	0/3 (0%)
Aiming		Aadland et al. [71] *SES* (3/3)	0/3 (0%)
Balance		de Waal and Pienaar [84] *No effect* (6/6)	0/6 (0%)
Running speed and agility	Jaakkola et al. [90] *SES* (1/24)	de Waal and Pienaar [84] *No effect* (6/6) Jaakkola et al. [90] *SES* (23/24)	1/30 (3.3%)
Leaping	Jaakkola et al. [90] *SES* (14/24) Jaakkola et al. [90] *MES* (2/24)	Jaakkola et al. [90] *SES* (8/24)	16/24 (66.7%)
Motor competence	Syväoja et al. [113] *SES* (2/2) Son and Meisels [112] *SES* (2/10) Son and Meisels [112] *LES* (2/10)	Ricciardi et al. [107] *SES* (8/8) Son and Meisels [112]^a^ (6/10) Chargas [81] *SES* 1/1	6/21 (28.6%)
	**Attention**		(I)
Balance	Zysset et al. [119] *SES* (1/1)	Niederer et al. [102] *SES* (2/2)	1/3 (33.3%)
Running speed and agility	Niederer et al. [102] *SES* (1/2)	Niederer et al. [102] *SES* (1/1)	1/2 (50%)
Motor competence	Zysset et al. [119] *SES* (1/1)		1/1 (100%)
	**Working memory**		(?)
Object manipulation		Osorio-Valencia et al. [105]^a^ (3/3) Capio et al. [80] *VSES-MES* (1/1)	0/4 (0%)
Locomotor skills		Osorio-Valencia et al. [105]^a^ (3/3) Capio et al. [80] *VSES-MES* (1/1)	0/4 (0%)
Balance	Niederer et al. [102] *SES* (2/4) Zysset et al. [119] *SES* (1/1) Osorio-Valencia et al. [105]^a^ (2/3)	Osorio-Valencia et al. [105]^a^ (1/3) Niederer et al. [102] *SES* (2/4)	5/8 (62.5%)
Running speed and agility	Niederer et al. [102] *SES* (2/4)	Niederer et al. [102] *SES* (2/4)	2/4 (50%)
Motor competence	Ludyga et al. [97] *MES* (4/8) Ludyga et al. [97] *LES* (2/8) Zysset et al. [119] *SES* (1/1)	Ludyga et al. [97] *MES* (2/8) Rigoli et al. [108] *SES* (2/2)	7/11 (63.6%)
	**Composite executive functions**		(I)
Object manipulation	MacDonald et al. [98] *MES* (1/1)		1/1 (100%)
Catching		Aadland et al. [71] *SES* (3/3)	0/3 (0%)
Aiming		Aadland et al. [71] *SES* (3/3)	0/3 (0%)
	**Cognitive functioning**		(I)
Object manipulation	Gu et al. [89] *SES* (1/2)	Gu et al. [89]^a^ (1/2)	1/2 (50%)
Locomotor skills	Gu et al. [89] *SES* (2/2)		2/2 (100%)
Balance	Zysset et al. [119] *SES* (1/1)		1/1 (100%)
Motor competence	Zysset et al. [119] *SES* (2/2)		1/1 (100%)
	**Psychosocial function**		(I)
Object manipulation	Gu et al. [89] *SES* (1/1)		1/1 (100%)
Locomotor skills	Gu et al. [89] *SES* (1/1)		1/1 (100%)
	**Social behavior**		(I)
Object manipulation	MacDonald et al. [98] *SES* (6/6)		6/6 (100%)
**Studies classified by motor competence outcome**			
	**Balance**		(I)
Academic performance		de Waal and Pienaar [84] *No effect* (6/6)	0/6 (0%)
	**Running speed and agility**		(I)
Academic performance	Jaakkola et al. [90] *SES* (3/8)	Jaakkola et al. [90] *SES* (5/8) de Waal and Pienaar [84] *No effect* (6/6)	3/14 (21.4%)
	**Leaping**		(I)
Academic performance	Jaakkola et al. [90] *SES* (5/8)	Jaakkola et al. [90] *SES* (3/8)	5/8 (62.5%)
	**Motor competence**		(I)
Academic performance	Syväoja et al. [113] *SES* (1/2)	Syväoja et al. [113] *SES* (1/2)	1/2 (50%)
	**Balance**		(I)
Attention	Zysset et al. [119] *SES* (1/1)		1/1 (100%)
	**Motor competence**		(I)
Attention	Zysset et al. [119] *MES* (1/1)		1/1 (100%)
	**Motor competence**		(I)
Working memory	Rigoli et al. [108] *SES* (2/4) Zysset et al. [119] *SES* (1/1)	Rigoli et al. [108] *SES* (2/4)	3/5 (60%)
	**Balance**		(I)
Cognitive functioning		Zysset et al. [119] *SES* (2/2)	0/2 (0%)
	**Motor competence**		(I)
Cognitive functioning	Zysset et al. [119] *SES* (2/2)		2/2 (100%)

Using the percentage score for each specific association for an outcome (i.e., catching – academic performance, balance – academic performance), the collective influence of these variables on the outcome was collated into a single percentage score and classified as either no association (0–33%), written as (0); indeterminate/inconsistent (34–59%), written as (?); or a positive ( +) or negative ( −) association (≥ 60%). When four or more studies found an association, it was classified as (+ +) or (− −) accordingly. If there were fewer than three studies in the domain, the strength of evidence was considered insufficient (I) to classify. Any study that included multiple analyses (> 8) pertaining to the same path (i.e., object manipulation to working memory) was not included in the results synthesis

Where adjusted values are used to report significance in studies, these are presented

^a^Effect size could not be calculated due to lack of information

*LES* large effect size, *MES* moderate effect size, *SES* small effect size

In studies investigating the relationship of motor competence and specific and composite-level executive functions, some supportive evidence was presented. Working memory was the most commonly assessed outcome, with balance (5/8 analyses), running speed and agility (2/4 analyses), and composite motor competence (7/11 analyses) all found to have a positive relationship, with effect sizes ranging from small to large [97, 102, 105]. For attention and composite executive functions, the evidence was considered insufficient, although single studies did find object manipulation competence to have a moderate relationship with composite executive functions [98] and balance, running speed, and agility, and composite motor competence to be positively associated with attention (small effect size) [102, 119]. Evidence for the reverse path (executive functions–motor competence) was considered similarly insufficient, with this being analyzed in only two studies [108, 119], and 7/11 analyses showing a small positive relationship between working memory and attention with later composite motor competence and dynamic balance. Although individual confounder variables (i.e., age, sex, BMI) were found to moderate the relationship between motor competence and executive functions (working memory and attention) in single studies [102, 108], collectively, the studies did not present a consistent pattern of evidence. In summary, there is some supportive evidence for the relationship between motor competence and academic performance and specific executive functions, with this especially true for working memory in pre-adolescent children. However, the level of evidence across all domains remains insufficient at this stage, with further studies needed.

#### Motor Competence and Social-Emotional Development

Only two observational studies assessed the longitudinal association of motor competence and aspects of social-emotional development [89, 98]. Although collectively, the level of evidence was deemed insufficient, there was supportive evidence presented in single studies. Specifically, for social behavior, object manipulation was found to be positively associated with the outcome in all analyses (6/6 analyses) in a single study [98]. In relation to psychosocial functioning, the role of object manipulation and locomotor skills was supported in single analyses [89]. For the studies that found a positive association between motor competence and psychosocial function and social behavior, process-oriented assessments of motor competence were used. It was not possible to identify an age- or sex-related influence on the relationship of motor competence and social-emotional development.

In summary, the available observational evidence suggests that motor competence may have an important relationship with social-emotional outcomes, but the level of evidence is insufficient and further studies are required to firstly identify domain-specific relationships and secondly, the potential role of moderating variables; see Tables S1–S3 in the electronic supplementary material (ESM) for observational evidence specific to age classification.

### Experimental Evidence

#### Motor Competence and Cognition

Five experimental studies (Table 6) [73, 75, 79, 87, 103] assessed the role of an intervention in eliciting positive adaptions in aspects of motor competence and academic performance, with three of these studies reporting an aligned improvement in object manipulation skills and aspects of academic performance in pre-school and pre-adolescent children [73, 74, 103]. Of the studies assessing pre-literacy skills as an outcome [73–75, 79, 85, 86], significant differences between intervention and control groups at follow-up were found in two studies [74, 86], whilst two further studies [73–75] showed a parallel improvement in motor competence and pre-literacy skills/academic performance. However, the study of Bedard et al. [75] did not include a control group, and although significant changes were found in motor and pre-literacy outcomes from pre- to post-intervention, no significant changes remained between post-intervention and follow-up. No studies investigated the causal relationship of motor competence with pre-literacy skills and academic performance outcomes.

**Table 6:** Analyses and results (experimental studies); studies using causal analyses are highlighted

Motor competence and cognitive and social-emotional outcomes	Significant causal improvement in IG (Reported effect sizes)	No significant causal improvement in IG (Reported effect sizes)	Summary of results (Analyses reporting a significant improvement/total analyses)	Significant aligned improvement in IG (Reported effect sizes)		No significant aligned improvement in IG (Reported effect sizes)	Significant difference between IG and CG post-intervention (Reported effect sizes)	No significant difference between IG and CG post-intervention (Reported effect sizes)
**Summary of studies classified by motor competence outcome**								
**Pre-literacy skills, academic performance, intellectual functioning**								
Object manipulation				Battaglia et al. [73]^a^ Battaglia et al. [73] Nobre et al. [103] *SES-LES* Nobre et al. [103] *LES*	Nobre et al. [103] VSES-*LES*		Battaglia et al. [74] *LES*	Battaglia et al. [73]^a^
Upper limb coordination					Botha and Africa [79] *SES*			Botha and Africa [79]^a^
Locomotor skills				Battaglia et al. [73]^a^ Battaglia et al. [74] *LES* Nobre et al. [103] *SES-LES* Nobre et al. [103] *LES*	Nobre et al. [103] VSES-*LES*		Battaglia et al. [74] *LES*	Battaglia et al. [73]
Balance					Botha and Africa [79] *MES*			Botha and Africa [79]^a^
Running speed and agility					Botha and Africa [79] *MES*			Botha and Africa [79]^a^
Motor competence				Bedard et al. [75] *SES* Bedard et al. [75] *LES* Battaglia et al. [74] *LES* Duncan et al. [86] *LES* Nobre et al. [103] *SES-LES* Nobre et al. [103] *LES*	Bedard et al. [75]* SES* Bedard et al. [75]* MES* Derman et al. [85] *No effect* Nobre et al. [103] *VSES-LES*		Battaglia et al. [74] *LES* Duncan et al. [86] *LES*	Ericsson [87] *MES* Ericsson [87]^a^ Derman et al. [85] *No effect*
**Composite executive functions**								
Balance	**Vazou et al. **[117] **(2/6 cool executive functions) *MES-LES***	**Vazou et al. **[117] **(4/6 cool executive functions) *SES*** **Vazou et al. **[117] **(hot executive functions) *SES***	2/5 **(40%)**	Vazou et al. [117] *SES-LES*	Katanić et al. [92] *SES*			Katanić et al. [92] *SES*
Motor competence							Aadland et al. [72]^a^ Condello et al. [82] (hot executive functions) *VSES*	Aadland et al. [72]^a^
**Cognitive functioning**								
Object manipulation				Boat et al. [78] *SES-VLES* Magistro et al. [99] *LES-VLES*	Lee et al. [94]^a^ Tseng et al. [116] *SES-MES*			Lee et al. [94]^a^
Locomotor skills				Boat et al. [78] *SES-MES* Magistro et al. [99] *LES-VLES*	Lee et al. [94]^a^			Lee et al. [94]^a^
Balance					Katanić et al. [92] *SES* Tseng et al. [116] *SES-MES*		Katanić et al. [92] *SES*	
Motor competence				Oppici et al. [104] (low cognitive) *SES-LES* Boat et al. [78] *SES-VLES* Magistro et al. [99] *LES-VLES*	Lee et al. [94]^a^ Biino et al. [77] *MES* Oppici et al. [104] (high cognitive) *SES-LES* Rudd et al. [111] (creative dance) *SES* Rudd et al. [111] (choreography dance) *SES*		Lee et al. [94]^a^	
**Creativity**								
Object manipulation	**Tocci et al. **[115] ***VSES-VLES***	**Tocci et al. **[115] ***VSES-VLES*** **Tocci et al. **[115] ***VSES-VLES***	1/3 **(33%)**	Rodríguez-Negro et al. [110] (GBP) *SES*	Rodríguez-Negro et al. [110] (BIP) *VSES* Rodríguez-Negro et al. [110] (DLP) *VSES-MES*			
Balance		**Tocci et al. **[115]^**a**^ **Tocci et al. **[115]^**a**^ **Tocci et al. **[115]^**a**^	0/3 **(0%)**	Rodríguez-Negro et al. [110] (GBP) *SES-LES*	Rodríguez-Negro et al. [110] (BIP) SES*-MES* Rodríguez-Negro et al^.^ [110] (DLP) *SES-MES*			
**Attention**								
Object manipulation				Rodríguez-Negro et al. [110] (GBP) *SES-MES*	Pesce et al. [106] *LES* Pesce et al. [106] *MES* Rodríguez-Negro et al. [110] (BIP) *VSES-SES* Rodríguez-Negro et al. [110] (DLP) *VSES-MES*		Pesce et al. [106] *LES* Pesce et al. [106] *MES*	
Balance		**Vazou et al. **[117]	0/1 **(0%)**	Rodríguez-Negro et al. [110] (GBP) *SES*-LES Vazou et al. [117] (RG) *SES*	Rodríguez-Negro et al. [110] (BIP) *SES-MES* Rodríguez-Negro et al. [110] (DLP) *SES-MES* Pesce et al. [106] *LES*		Pesce et al. [106] *LES* Pesce et al. [106] *MES*	
Motor competence							Ericsson [87]^a^	Ericsson [87]^a^
**Working memory**								
Object manipulation	**Tocci et al. **[115]^**a**^		1/1 **(100%)**	Lin et al. [96] (response accuracy) *MES* Lin et al. [96] (Reaction time) *SES* Zhang et al. [118] *LES*	Lin et al. [96] (tCNV)^a^		Lin et al. [96] (Response accuracy) *MES* Lin et al. [96] (iCNV) *MES*	Lin et al. [96] (Reaction time) *SES* Lin et al. [96] (tCNV)^a^
Balance		**Tocci et al. **[115]^**a**^	0/1 **(0%)**	Lin et al. [96] (Response accuracy) *MES-LES* Lin et al. [96] (Reaction time) *SES* Zhang et al. [118] *SES-LES*			Lin et al. [96] (Response accuracy) *MES-LES* Zhang et al. [118] *SES* Zhang et al. [118] *SES*	Lin et al. [96] (Reaction time) *SES*
Motor competence	**Oppici et al. **[104] ***SES-MES***	**Oppici et al. **[104] ***MES***	1/2 **(50%)**	Koutsandréou et al.^.^[93]* LES* Rudd et al. [111] (Choreography dance) *SES-MES* Oppici et al. [104] (High cognitive) *SES*	Pesce et al. [106] *SES* Condello et al. [75] *SES* Rudd et al. [111] (Creative dance) *SES* Oppici et al. [104] (Low cognitive) *SES*		Koutsandréou et al. [93]* SES* Biino et al. [77] *LES*	Koutsandréou et al. [93] *SES* Katanić et al. [92] *MES*
**Inhibition**								
Ball skills	**Pesce et al. **[106] ***SES*** **Tocci et al. **[115] ***MES-VLES***		2/2 **(100%)**				Pesce et al. [106] *SES*	
Balance		**Tocci et al. **[115]^**a**^	0/1 **(0%)**				Pesce et al. [106] *SES*	
Motor competence				Condello et al. [82] *MES* Rudd et al. [111] (Choreography dance) *SES-MES*	Biino et al. [77] *SES-LES* Oppici et al. [104] (High cognitive) *VSES-LES* Oppici et al. [104] (High cognitive) *VSES-LES* Rudd et al. [111] (Creative dance) *SES*			Li et al. [95] *VSES-SES*
**Impulse control**								
Balance		**Vazou et al. **[117]	0/1 **(0%)**		Rodríguez-Negro et al. [110] (BIP) *VSES-SES* Rodríguez-Negro et al. [110] (DLP) *SES* Rodríguez-Negro et al. [110] (GBP) *VSES-LES* Vazou et al. [117] *MES-LES*			Rodríguez-Negro et al. [110] (BIP) V*SES-SES* Rodríguez-Negro et al. [110] (DLP) *SES* Rodríguez-Negro et al. [110] (GBP) *VSES-LES* Vazou et al. [117] *MES-LES*
Object manipulation					Rodríguez-Negro et al. [110] (BIP) *VSES-SES* Rodríguez-Negro et al. [110] (DLP) *SES* Rodríguez-Negro et al. [110] (GBP) *VSES-SES*			Rodríguez-Negro et al. [110] (BIP) *VSES-SES* Rodríguez-Negro et al. [110] (DLP) *SES* Rodríguez-Negro et al. [110] (GBP) *VSES-SES*
Motor competence							Ericsson [87]^a^	Ericsson [87]^a^
**Behavioral regulation**								
Motor competence					Mulvey et al. [101] *MES* Robinson et al. [109] *MES*		Mulvey et al. [101] *MES* Robinson et al. [109] *MES*	
**Social-emotional skills**								
Object manipulation				Jalilnasab et al. [91] *LES*	Berleze and Valentini [76] *LES-VLES*		Berleze and Valentini [76] *LES-VLES* Jalilnasab et al. [91] *LES*	Minghetti et al. [100] *VSES*
Locomotor skills				Jalilnasab et al. [91] *LES*	Berleze and Valentini [76] *LES-VLES*		Berleze and Valentini [76] *LES-VLES* Jalilnasab et al. [91] *LES*	Minghetti et al. [100] *VSES*
Balance				Fathirezaie et al. [88]^a^			Fathirezaie et al. [88]^a^	
Running speed and agility				Fathirezaie et al. [88]^a^			Fathirezaie et al. [88]^a^	
Motor competence	**Condello et al. **[82] **(Enriched PE) *SES***	**Condello et al. **[82] ***SES***	1/2 **(50%)**	Condello et al. [82] *SES* Jalilnasab et al. [91] *LES*	Condello et al. [82] *SES* Derman et al. [85] *No effect*		Condello et al. [82] *SES* Jalilnasab et al. [91] *LES*	Condello et al. [82] *SES* Derman et al. [85] *No effect* Minghetti et al. [100] *VSES*
**Summary of studies classified by motor competence outcome**								
**Catching**								
Intellectual functioning					De Oliveira et al. [83] *SES*			De Oliveira et al. [83] *SES*
**Balance**								
Intellectual functioning					De Oliveira et al. [83] *SES*			De Oliveira et al. [83] *SES*
**Object manipulation**								
Temperament				Taunton et al. [114] *LES*			Taunton et al. [114] *MES*	
**Locomotor skills**								
Temperament				Taunton et al. [114] *LES*			Taunton et al. [114] *LES*	

*BIP* balance intervention program, *CG* control group*, DLP* drama learning program, *GBP* game-based program, *iCNV* initial contingent negative variation, *IG* intervention group*, LES* large effect size, *MES* moderate effect size, *PE* physical education, *PEG* physical education group, *RG* rhythmic group, *SES* small effect size, *tCNV* terminal contingent negative variation*, VLES* very large effect size, *VSES* very small effect size

A further five studies [82, 104, 106, 115, 117] included analyses of how a change in motor competence influenced or mediated adaptions in executive functions. Two studies presented causal findings, with evidence of a significant causal improvement found for cold executive functions (2/5 analyses) [117], working memory, and inhibition [115]. Taken together, the studies failed to provide consistent supportive evidence for outcomes in pre-adolescent and adolescent children, with no clear evidence of a heightened relationship of motor competence with either ‘hot’ or ‘cold’ executive functions. Similarly, inconsistent findings were presented in two studies that tested the mediating role of motor competence on the influence of a PE intervention on executive functions [82, 106]. In addition, 14 studies [72, 77, 78, 83, 87, 92–94, 96, 99, 110, 111, 116, 118] analyzed outcomes of motor competence and cognitive functioning and executive functions (individual-level or composite) in parallel, with these largely focused on pre-adolescent children. Of the studies, nine found evidence of an aligned improvement in both motor competence and cognitive functioning and some or all executive functions. Despite a consistent pattern of improvement at a domain level not being evident, cognitive functioning, working memory, and attention were consistently found to have improved in multiple studies. Insight into the importance of the qualitative underpinning of an intervention to the relationship between motor competence and executive functions was provided in two studies, with these studies finding a fitness-targeted intervention to be far less influential on cognitive adaptions [92, 93]. Koutsandréou et al. [93] reported a higher post-intervention motor competence score in a motor-exercise group than found in a control group, and a higher gain in working memory performance in the motor-exercise group than both the control and a cardiovascular-exercise intervention group. Some further support is offered by the study of Oppici et al. [104], which also highlighted the influence of the underpinning cognitive demand within an intervention in promoting aligned improvements in motor competence and working memory.

In summary, too few experimental studies have investigated the causal relationship between motor competence and cognitive outcomes, or the moderating role of motor competence in the relationship of physical activity and cognitive development. Evidence from non-causal analytical approaches suggests that there is alignment in the development of motor and cognitive domains, with this most evident for cognitive functioning, working memory, and attention in pre-adolescent children.

#### Motor Competence and Social-Emotional Development

Ten experimental studies [76, 82, 85, 88, 91, 100, 101, 109, 114, 117] investigated outcomes associated with behavioral regulation and social-emotional development. Collectively, the included evidence is inconsistent at a domain level, although several studies found an aligned improvement in locomotor and balance skills and social-emotional outcomes in pre-school and pre-adolescent children. In pre-school children, supportive evidence was presented for the relationship with aspects of self-regulation, with effect sizes ranging from medium to very large [109, 114]. Further support for the role of locomotor skills and balance was provided in two pre-adolescent samples [76, 88]. Using mediation analysis, Condello et al. [82] found motor competence mediated the positive effect of an enriched PE intervention on peer-rated cooperation but not peer-rated empathy. At an individual level, some evidence found waist circumference and sex to act as moderators, while at an environmental level, outdoor, rather than within-classroom, learning was found to enhance the improvement of assessed outcomes.

In summary, there are insufficient experimental studies supporting the relationship between motor competence and social-emotional development. However, there is initial evidence to suggest that motor competence may play an important mediating role between physical activity and social-emotional outcomes and that this may be moderated by task, environmental, and individual characteristics; see Tables S4–S6 in the ESM for experimental evidence specific to age classification.

## Discussion

This systematic review aimed to evaluate and summarize evidence pertaining to the longitudinal relationship between motor competence and cognitive and social-emotional outcomes. Notably, the review sought to establish the role of motor competence as a mechanism through which physical activity may support chronic cognitive and social-emotional adaptions in children and adolescents, while considering individual, task-related, and environmental moderators of these relationships.

Overall, observational evidence supporting the positive influence of motor competence on cognitive and social-emotional outcomes was indeterminate and insufficient for all age classifications, with many studies considered to have poor internal and external validity. Few observational studies investigated the reverse path (cognitive and social-emotional development to motor competence), with those studies that did presenting similarly indeterminate evidence. Whilst individual studies do provide some indication of a relationship and warrant discussion, the current review highlights key issues that currently contribute to the inconclusive evidence base. Unfortunately, for many of the included studies, the primary analyses were not deemed eligible for inclusion in this review, as the studies had used a single composite measure of motor competence that included fine and gross motor skills. An essential aim of this review was to identify which processes are interrelated at a construct (motor competence) and domain level (cognitive and social-emotional development).

Several experimental studies provided evidence for the positive association between motor competence and cognitive and social-emotional development, along with identifying the mechanistic pathways that may underpin this. Specifically, there is some support for associated gains in motor competence and cognition or social-emotional outcomes following cognitively enriched physical activity interventions in pre-adolescent children [93, 104, 115]. However, evidence relating to the role of specific constructs of motor competence (e.g., object manipulation) remains largely indeterminate, although multiple studies did present support for a heightened role of object manipulation skills in pre-adolescence [78, 96, 99, 106, 115]. The lack of methodological alignment between individual studies (e.g., 23 different motor competence assessments were used across the 49 included studies), and the failure of studies to adequately capture the contextual influence of the intervention [28], also make it difficult to identify common themes. The inclusion criteria for the current review permitted studies that assessed parallel gains in motor competence and cognitive and social-emotional development. This type of study design cannot explicitly answer the question of whether changes in motor competence have a causal influence upon outcome variables (and vice versa), unless the association in motor and cognitive gains is evaluated with correlational or mediational analyses and emerges only, or is more pronounced, for the intervention group. To date, this type of approach is rare [6, 29, 138] and warrants future, appropriately tailored, research.

### Motor Competence to Cognition

#### Pre-Literacy Skills, Academic Performance, and Intellectual Functioning

The observational evidence was indeterminate and insufficient for both path directions (i.e., motor competence to pre-literacy skills, academic performance, intellectual functioning; and pre-literacy skills, academic performance, intellectual functioning to motor competence), with no consistent domain-specific or age-related relationships identified. This is in line with the earlier systematic review of van der Fels and colleagues [17], which found similarly inconsistent evidence for comparable outcomes. In adolescents, there was no clear evidence of a positive influence of motor competence on subject-specific and overall academic performance, which was also true for analyses relating to the reverse path [90, 113]. Of these studies, only one study [90] included domain-specific analyses. The leaping skill was found to be the strongest predictor of global academic performance (small to moderate effect sizes), but similar support was not evident for an academic subject-specific relationship. It is hypothesized that leaping, together with tasks such as galloping and sliding, may still not be sufficiently automatized during adolescence and may place greater emphasis on the executive functions that are shown to strongly align with academic performance [25]. Despite some previous supporting evidence [139], age was not found to be a moderator of pre-literacy skills, academic performance, and intellectual functioning in the observational evidence in the current review. As few studies included executive functions as moderators of academic outcomes, the observational results of this review cannot build on prior evidence that has suggested an increased specificity, with age, in the cognitive abilities associated with individual academic subjects [32]. To advance understanding, there is a need for studies to perform construct-level and subject-specific analyses. It is also important that studies consider the inclusion of executive functions (i.e., processing speed, working memory) as moderators, in an attempt to identify the mechanisms through which constructs of motor competence may influence individual subject performance [32].

As previously noted, much research investigating the relationship between motor competence and academic performance has focused on pre-adolescent samples, specifically pre-school children and those transitioning into school. Only one observational study [84] analyzed the relationship in both directions in pre-adolescent children, finding no significant relationship between balance, running and agility, and several academic domains. Whilst at similar ages composite motor competence was found to influence reading and maths performance (small to large effect sizes), it is important to consider that in results not reported in the current review [79, 84], fine motor skills were found to be of greater influence than motor competence on the assessed academic disciplines. In pre-school children, acknowledging the role of fine motor skills may be important, as visual-motor integration is proposed to have a key influence on many of the academic activities that children participate in, including reading, handwriting and letter-word identification [25, 140–142].

In contrast to the observational study evidence, the included experimental studies provided evidence of a developmental relationship between motor competence and pre-literacy skills, along with the underpinning mechanisms that may support this. Yet, there was no clear pattern of divergence in the role of individual constructs of motor competence on improved academic performance, with a single study showing a positive role of object manipulation and locomotor skills in pre-adolescent children [115]. Battaglia and colleagues [74] built on the preliminary findings of their earlier study [73] and found locomotor and object manipulation skills correlated significantly with pre-literacy skills following a PE program intervention. In pre-school children, a key underpinning factor in the efficacy of a PE intervention in improving motor and pre-literacy skills could be the interactions with peers and the demand on visual-motor integration abilities [74]. Bedard et al. [75] and Duncan et al. [86] also found intervention-related improvements in motor competence and pre-literacy skills, although in the study of Bedard and colleagues [75], post-hoc analyses found these improvements diminished upon completion of the intervention and at follow-up. This study also had a small sample size, did not include a control group, and reported poor adherence to some aspects of the intervention (e.g., at-home practice).

Only one study [83] analyzed the intellectual functioning to motor competence path. Moreover, the study of De Oliveira et al. [83] is the only one that investigated the moderating influence of intellectual functioning, reporting that improvements in motor competence following a within-school intervention occurred, irrespective of participant intellectual functioning score pre-intervention [83]. The failure of intellectual functioning to moderate improvements in motor competence may highlight the less distinct formation of executive functions in preschool-aged children, although methodological limitations warrant consideration [143, 144]. Taken together, the level of experimental evidence relating to indicators of academic performance was undermined by a lack of rigor in assessing the potential role of the interventional components [87].

In summary, there exists initial evidence supporting the role of task characteristics (e.g., qualitative physical activity) and to a lesser extent, environmental characteristics (e.g., school), in influencing the motor competence to pre-literacy skills path at pre-school age. Some evidence is presented to support the direct path of motor competence to academic performance, although this is not consistent and warrants further investigation, using construct and subject-specific analyses. Indeed, across all ages, more studies are necessary, especially those that target pre-adolescent and adolescent children. There remains insufficient evidence for the moderating role of individual characteristics (e.g., sex and BMI).

#### Cognitive Functioning and Executive Functions

The current review presents indeterminate observational evidence supporting the relationship between motor competence and cognitive functioning and executive functions, with only two studies including analyses of the influence of cognitive functioning or executive functions on motor competence [108, 119]. Collectively, these studies failed to present a consistent influence for a specific construct of motor competence, with balance, running speed, and composite motor competence all being found to positively influence working memory, composite executive functions, and cognitive functioning. It has previously been purported that locomotor skills are more influential on working memory and that object manipulation skills have a greater influence on inhibition [145]. However, too few studies have empirically investigated these construct- and domain-specific relationships. Several factors may contribute to the heterogeneity found in the study results in this review. First, there are notable inter-study differences in the tasks used to measure executive functions and the methods used for motor competence assessment (i.e., process- or product-oriented); for example, whether the assessment of motor competence sufficiently challenges the children to develop movement solutions, and for the assessment of executive functions, whether there is specificity to the intended executive function (i.e., working memory) or whether the assessments are engaging additional processes [27, 146]. Second, many of the studies include children of pre-school ages, for whom it is proposed executive functions are less defined [144, 146]. To the best of our knowledge, evidence of the relationship of motor competence to cognitive and social-emotional development in adolescents is less frequent, or pre-adolescent and adolescent samples are combined [147] and mainly cross-sectional in nature, thus limiting insight into causal inference [148–150].

There is some encouraging experimental evidence supporting the role of cognitively enriched physical activity interventions in the development of motor competence and both working memory and inhibitory control. Whilst less consistent, further evidence was found at a composite level (motor competence and composite executive functions), although little evidence was found to support a similar influence on the development of cognitive flexibility. There is an apparent greater focus on ‘cool’ executive functions (i.e., elicited in neural conditions, and including cognitive flexibility, inhibition) in the literature, despite ‘hot’ executive functions (i.e., goal-directed processes that include emotion and motivation) being suggested to be strongly aligned to the development and execution of motor skills in cognitively enriched environments [38, 151]. The content, context, and fidelity to the intervention delivery appear key to further understanding the underpinning mechanisms of the motor competence–executive functions relationship. For example, in the study of Aadland and colleagues [72], the analyses revealed significant effects of the intervention (increased within-school physical education, physical activity homework) on motor competence and composite executive functions score, without a similar effect on levels of physical activity [72]. These results can likely be attributed to the development of motor competence within an enriched physical activity context, and not solely through the neurotrophic hypothesis, according to which physiological adaptions associated with quantitative physical activity levels are viewed as the key causal mechanism [31, 72]. This hypothesis is further supported by the study of Koutsandréou et al. [93], who despite finding similar between-group (motor competence vs cardiovascular-focused) improvements in motor competence, found a greater improvement in working memory in the motor competence group. Additional experimental support for the path of motor competence and executive functions is offered by Pesce et al. [106] and Tocci et al. [115]. Pesce et al. [106] found ball skill competence mediated the influence of an enriched PE intervention (directed exploration, task complexity) on inhibitory function, with this mediated path subsequently moderated by the level of outdoor play. While Pesce et al. [106] failed to find a similar influence on attention and working memory updating, the study found a causal relationship (motor competence–executive functions) and identified a heightened role of object control skills in promoting positive cognitive development. Reporting the relationship between motor competence and executive functions as potentially developmental and domain specific, these experimental studies do offer some support to accumulating evidence provided by cross-sectional studies [17, 145, 152, 153]. Moving forward, future experimental studies must emphasize pedagogy fidelity as a key assessment approach [111]. Given there is now a growing agreement that motor competence and executive functions can be promoted in learning contexts that integrate cognitively challenging, complex, and novel tasks [154], it is crucial researchers consider the specific executive functions they are targeting, as well as the mechanisms for change that will underpin this within an intervention [111].

In summary, some support exists for the path of motor competence and executive functions, most notably for the direct paths of working memory and inhibition. While it appears that this relationship with individual domains (i.e., working memory and inhibition) is more apparent in school-aged children, further studies are needed to confirm this. There remains insufficient evidence for the path of motor competence and cognitive flexibility. Likewise, there is similarly insufficient evidence supporting a clear influence of individual constructs of motor competence, although the included results do present a heightened role of object manipulation skills for some aspects of executive function. The experimental evidence does support the crucial role of task characteristics (e.g., cognitively enriched physical activity) in influencing outcomes of motor competence and executive functions, although more consistent reporting of intervention fidelity is needed. Finally, too few studies have considered the moderating role of sex and BMI in their analyses and, as such, there remains indeterminate evidence.

### Motor Competence to Social-Emotional Development

#### Social-Emotional Development

As with those studies investigating cognitive outcomes, the important role of task, environmental, and individual characteristics (as identified in our conceptual model) is supported in several experimental studies. For example, Berleze and Valentini [76] highlighted the effectiveness of a mastery motivational program not only for positively influencing motor competence and social acceptance in obese low socio-economic status children, but also for influencing the daily routine of children (e.g., reduced time spent watching television). Indeed, a crucial mechanism in interventions aiming to promote motor and social-emotional development may be the level of autonomy provided to the children and the incorporation of a holistic, whole-child approach, as opposed to a lone pedagogical stance [82]. Aligned to the promotion of autonomy is the potential role of the environmental context, specifically ‘affordances’ [155]. The findings of Fathirezaie et al. [88] support a greater emphasis on natural environments, where children can explore and develop play behaviors through interactions with a diverse set of affordances. Furthermore, for younger children, such autonomous contexts may promote verbal interactions and facilitate social and communication skill development [91, 156].

Taken together, there was relatively consistent and supportive evidence for the motor competence to social-emotional development path. Whilst this relationship was primarily assessed in experimental studies that did not directly analyze a causal influence, the evidence does provide some agreement with the results of earlier cross-sectional studies and those completed in a clinical setting [157, 158]. It is hypothesized that the influence of motor competence on social-emotional development is apparent from early childhood, with poor motor competence contributing to difficulties in the social domain (i.e., social isolation, fewer peer interactions), and these difficulties potentially leading to the development of coping strategies, such as avoiding more physically active pursuits [159–162]. Gu et al. [89] support this hypothesis, as motor competence was found to influence psychosocial development. At younger ages especially, a plausible mechanism may be that a high level of motor competence promotes a positive participation cycle, whereby children become more immersed in opportunities that promote social-emotional skill development [163]. It is also suggested that object manipulation skills may have a greater influence on this positive participation cycle as these underpin active play to a greater extent than locomotor and balance skills. There is some support for this in the included studies [89, 98].

The eligible studies that included pre-school aged children highlight that the social-emotional consequences of poor motor competence are apparent from young ages. Prior evidence has shown this relationship to exist as early as kindergarten (e.g., aged 3 years and younger) [164], and it is suggested that the strength of the relationship increases into adolescence as a consequence of consistent exposure to secondary stressors, along with a more prominent influence of mediating and moderating variables [157, 165]. Evidence that the relationship may be reciprocal was also provided in a study [114], where a more positive score in facets of temperament (baseline) was associated with greater improvement in motor competence post-intervention. However, this hypothesized relationship warrants further rigorous investigation, specifically the path of social-emotional development to motor competence, as it is proposed that it is motor competence that proceeds social-emotional development in children [166]. Moreover, social-emotional health is a key indicator of wider psychosocial health and academic behavior, along with a wider health identity, especially in adolescence where it is associated with dysfunctional behavior and poor mental health [167, 168].

In summary, there is some supportive evidence for the relationship between motor competence and aspects of self-control/regulation, cooperation, and composite social skills, which was found for pre-school and pre-adolescent children. However, there is insufficient evidence to assertively confirm a moderating role of age and sex. Moving forward, there is a need for more studies that include adolescent samples. Collectively, the studies also fail to present any clear construct-level relationships; with object manipulation, locomotor skills, balance, and composite motor competence being predictors of social-emotional development in individual studies. As with aspects of cognitive development, the moderating role of task and environmental characteristics is emphasized in the supportive experimental evidence. Specifically, the positive influence of cognitively enriched PE interventions promotes autonomy, stimulates interaction, and affords engagement with the environment.

### Strengths and Limitations

By synthesizing observational and experimental evidence, the current review has several key strengths and provides an important overview of the current evidence for all of the paths relating to the relationship of motor competence and cognitive and social-emotional development. This review is the first to present a synthesis of longitudinal observational and experimental evidence, with no applied date restriction, and including effect size calculations for all studies where possible. The review also highlights important considerations that should be addressed in future empirical research. Not including cross-sectional evidence has provided an opportunity to build a more precise interpretation of the developmental and domain-specific relationship between aspects of motor competence and cognitive and social-emotional development. Moreover, synthesizing experimental evidence affords the opportunity to understand the importance of the interaction between motor competence and contextual mechanisms on cognitive and social-emotional outcomes. Lastly, developing a conceptual model is a central component of this review and provides an underpinning representation of the key relationships through which research questions can be formulated and future research guided.

There are several review limitations that should be acknowledged. In attempting to develop a clearer understanding of the contextual influences that may exist on the relationship between observed outcomes, the authors included studies where the analysis of outcomes was completed in parallel. Despite providing scope for wider analysis by including experimental studies that assessed outcome changes individually, this approach must be considered as less than desirable when interpreting the statements included within this review. Moreover, the large variability in assessment methods and outcomes within the included studies made it difficult to make clear assertions as to the strength of evidence. Indeed, the high level of between-study heterogeneity within this review meant that meta-analyses were not possible. In addition, despite calculating the effect sizes for analyses where possible, the failure of several studies to report the required information limited full application of this. Lastly, the study eligibility criteria meant that many primary analyses were not always included, as they had analyzed motor competence and fine motor skills together as a single composite outcome. Therefore, many of the analyses reflect correlation analyses, which were not controlled for confounders.

### Future Directions

As highlighted in earlier systematic reviews [28, 169], there has been an exponential increase in primary studies investigating the role of chronic and acute physical activity in promoting positive cognitive development. Aligned to this, there has been a collective effort to better understand the position of motor competence as a key underpinning mechanism for this relationship. However, the evidence base remains indeterminate for many of the investigated domains. This is likely fostered by many studies lacking in methodological rigor, and failing to sufficiently report on the moderating and contextual factors that may, or may not, trigger mechanisms acting in the relationship between physical activity, motor competence, and cognitive and social-emotional outcomes [28, 170]. For experimental studies, greater emphasis must be directed towards ensuring thorough process evaluations are reported, providing researchers the opportunity to consistently identify those characteristics of an intervention that may prompt a causal or moderating influence [170]. It is also important that researchers display awareness of the ambiguity surrounding the measurement of cognitive constructs, together with ensuring that there is agreement between the measurement task used and the selected operational term [171]. For example, when assessing executive functions, a commonly cited challenge is whether multiple processes are in fact being assessed, such as verbal and motor responses, and whether this may be contributing to the inconsistent evidence [172]. Researchers must also work to limit threats to internal validity, such as the influence of using the same cognitive test at different time points, and acknowledge the potential role of natural cognitive maturation [171]. A further consideration for researchers is the ecological validity of selected motor competence assessments, and whether the instrument provides an opportunity for a robust understanding of the relationship between motor competence and cognitive and social-emotional outcomes. From an ecological perspective, it is hypothesized that the variability and constraints within a context underpin the associated development of executive functions and wider cognitive outcomes [41]. Therefore, motor competence assessments such as the Dragon Challenge or the CAMSA may afford a greater insight into these specific relationships than closed-skill assessments that present fewer performance-related constraints (i.e., TGMD-3, MABC) [2]. In addition, the large variety of motor competence assessments render comparative analysis difficult. Moreover, many studies have conducted their primary analysis using a composite-level measure of motor competence, which does not provide an opportunity to establish domain-specific influences. Future studies should ensure that construct-level motor competence is also included in primary analyses. Lastly, to understand how the trajectories of biological and cognitive maturity influence the relationship of these outcomes with advancing age and specific to sex, more studies including adolescent samples are needed where these moderating influences are accounted for within study designs. By investigating the influence of biological maturity and sex, such studies limit the potential confounding influence of studies pooling both sexes in their analyses and offer opportunity for further understanding of the non-linear relationships between motor and cognitive domains [27]. Similar to the recommendation of Lima and colleagues [13], it is important that future, longer-term studies aim to capture the developmental and causal relationships that may exist between the key components highlighted in their conceptual model and advanced upon in this systematic review.

## Conclusions

The authors present a conceptual model to promote research with a strong rationale and that can provide consideration of the contextual and developmental influences that moderate the relationship between motor competence and cognitive and social-emotional development. To date, too many studies have approached the role of motor competence in influencing cognitive and social-emotional outcomes from an exploratory position, without a clear consideration for the mechanisms underpinning their hypotheses. As such, there are high levels of study heterogeneity and the evidence base is difficult to synthesize, with conclusions remaining speculative. However, whilst acknowledging the limitations of the data presented, some supportive evidence for individual paths hypothesized in the conceptual model is presented within this review. Specifically, observational evidence supports the influence of motor competence on aspects of executive functions and academic performance, although clear patterns of domain-specific relationships are still not manifest. Whilst some experimental studies provide preliminary support for the alignment between the underlying processes responsible for executive functions (i.e., working memory) and those required to engage in enriched movement interventions, moving forward successfully, researchers must ensure their study design encompasses the moderating influences that will assist in further developing understanding within this field.

### Supplementary Information

Below is the link to the electronic supplementary material.

Supplementary file1 (DOCX 59 KB)
