# Supplementary material for: The Influence of Motor Competence on Broader Aspects of Health: A Systematic Review of the Longitudinal Associations Between Motor Competence and Cognitive and Social-Emotional Outcomes
Source: Sports Med. 2023 Nov 21;54(2):375–427. doi: 10.1007/s40279-023-01939-5 (PMC10933160; doi:10.1007/s40279-023-01939-5)
Supplement: Supplementary file 1 — Supplementary file1 (DOCX 59 KB) [file 40279_2023_1939_MOESM1_ESM.docx]

**Supplementary information**

**Electronic Supplementary Table S1.** Analyses and Results for pre-school children (Observational studies)

| **Motor competence and cognitive and social-emotional outcomes** | **Significant improvement**  (Reported effect sizes) | **No significant improvement**  (Reported effect sizes) | **Summary of results**  (Analyses reporting a significant improvement/total analyses) |
| --- | --- | --- | --- |
|  | **Studies classified by cognitive and social-emotional outcome** | | |
|  | Academic performance **(I)** | | |
| Motor competence | Son and Meisels [112] *SES* (2/10)  Son and Meisels [112] *LES* (2/10) | Son and Meisels [112]^*^ (6/10) | 4/10 **(40%)** |
|  | Attention **(I)** | | |
| Balance | Zysset et al. [119] *SES* (1/1) |  | 1/1 **(100%)** |
| Motor competence | Zysset et al. [119] *SES* (1/1) |  | 1/1 **(100%)** |
|  | Working memory **(I)** | | |
| Object manipulation |  | Osorio-Valencia et al. [105]* (3/3)  Capio et al. [80] *VSES-MES* (1/1) | 0/4 **(0%)** |
| Locomotor skills |  | Osorio-Valencia et al. [105]* (3/3)  Capio et al. [80] *VSES-MES* (1/1) | 0/4 **(0%)** |
| Balance | Zysset et al. [119] *SES* (1/1)  Osorio-Valencia et al. [105]* (2/3) | Osorio-Valencia et al. [105]* (1/3) | 3/4 **(75%)** |
|  | Composite executive functions **(I)** | | |
| Object manipulation | MacDonald et al. [98] *MES* (1/1) |  | 1/1 **(100%)** |
|  | Social behavior **(I)** | | |
| Object manipulation | MacDonald et al. [98] *SES* (6/6) |  | 6/6 **(100%)** |
|  | **Studies classified by motor competence outcome** | | |
|  |  | Balance | **(I)** |
| Attention | Zysset et al. [119] *SES* (1/1) |  | 1/1 **(100%)** |
|  | Motor competence **(I)** | | |
| Attention | Zysset et al. [119] *MES* (1/1 ) 1/1 **(100%)** | | |
|  | Motor competence **(I)** | | |
| Working memory | Zysset et al. [119] *SES* (1/1) |  | 1/1 **(100%)** |
|  |  | Balance | **(I)** |
| Cognitive functioning |  | Zysset et al. [119] *SES* (2/2) | 0/2 **(0%)** |
|  |  | Motor competence | **(I)** |
| Cognitive functioning | Zysset et al. [119] *SES* (2/2) |  | 2/2 **(100%)** |

*ES* effect size could not be calculated due to lack of information, *LES* large effect size, *MES* moderate effect size, *SES* small effect size, *VSES* very small effect size

Where adjusted values are used to report significance in studies these are presented

Using the percentage score for each specific association for an outcome (i.e., catching – academic performance, balance – academic performance), the collective influence of these variables on the outcome was collated into a single percentage score and classified as either no association (0–33%), written as ‘0’; indeterminate/inconsistent (34–59%), written as ‘?’; or a positive ‘+’ or negative ‘−’ association (≥ 60%). If there were fewer than three studies in the domain, the strength of evidence was considered insufficient (I) to classify. Any study that included multiple analyses (>8) pertaining to the same path (i.e., object manipulation to working memory) was not included in the results synthesis.

**Electronic Supplementary Table S2.** Analyses and Results for pre-adolescent children (Observational studies)

| **Motor competence and cognitive and social-emotional outcomes** | **Significant improvement**  (Reported effect sizes) | **No significant improvement**  (Reported effect sizes) | **Summary of results**  (Analyses reporting a significant improvement/total analyses) |
| --- | --- | --- | --- |
|  | **Studies classified by cognitive and social-emotional outcome** | | |
|  | Academic performance **(I)** | | |
| Balance |  | de Waal and Pienaar [86]  *No effect* (6/6) | 0/6 **(0%)** |
| Running speed & agility |  | de Waal and Pienaar [86] *No effect* (6/6) | 0/6 **(0%)** |
| Motor competence |  | Ricciardi et al. [109] *SES* (8/8) | 0/8 (**0%)** |
|  | Attention **(I)** | | |
| Balance |  | Niederer et al. [104] *SES* (2/2) | 1/3 **(33.3%)** |
| Running speed & agility | Niederer et al. [104] *SES* (1/2) | Niederer et al. [104] *SES* (1/1) | 1/2 **(50%)** |
|  | Working memory **(?)** | | |
| Balance | Niederer et al. [104] *SES* (2/4) | Niederer et al. [104] *SES* (2/4) | 2/4 **(50%)** |
| Running speed & agility | Niederer et al. [104] *SES* (2/4) | Niederer et al. [104] *SES* (2/4) | 2/4 **(50%)** |
| Motor competence |  | Rigoli et al. [110] *SES* (2/2) | 1/3 **(33.3%)** |
|  | Cognitive functioning **(I)** | | |
| Object manipulation | Gu et al. [91] *SES* (1/2) | Gu et al. [91]* (1/2) | 1/2 **(50%)** |
| Locomotor skills | Gu et al. [91] *SES* (2/2) |  | 2/2 **(100%)** |
|  | Psychosocial function **(I)** | | |
| Object manipulation | Gu et al. [91] *SES* (1/1) |  | 1/1 **(100%)** |
| Locomotor skills | Gu et al. [91] *SES* (1/1) |  | 1/1 **(100%)** |
|  | **Studies classified by motor competence outcome** | | |
|  | Balance **(I)** | | |
| Academic performance |  | de Waal and Pienaar [86] *No effect* (6/6) | 0/6 **(0%)** |
|  | Running speed & agility **(I)** | | |
| Academic performance |  | de Waal and Pienaar [86] *No effect* (6/6) | 0/6 **(0%)** |
|  | Motor competence **(I)** | | |
| Working memory | Rigoli et al. [110] *SES* (2/4) | Rigoli et al. [110] *SES* (2/4) | 2/4 **(50%)** |

*ES* effect size could not be calculated due to lack of information, *LES* large effect size, *MES* moderate effect size, *SES* small effect size

Where adjusted values are used to report significance in studies these are presented

Using the percentage score for each specific association for an outcome (i.e., catching – academic performance, balance – academic performance), the collective influence of these variables on the outcome was collated into a single percentage score and classified as either no association (0–33%), written as ‘0’; indeterminate/inconsistent (34–59%), written as ‘?’; or a positive ‘+’ or negative ‘−’ association (≥ 60%). If there were fewer than three studies in the domain, the strength of evidence was considered insufficient (I) to classify. Any study that included multiple analyses (>8) pertaining to the same path (i.e., object manipulation to working memory) was not included in the results synthesis.

**Electronic Supplementary Table S3.** Analyses and Results for adolescents (Observational studies)

| **Motor competence and cognitive and social-emotional outcomes** | **Significant improvement**  (Reported effect sizes) | **No significant improvement**  (Reported effect sizes) | **Summary of results**  (Analyses reporting a significant improvement/total analyses) |
| --- | --- | --- | --- |
|  | **Studies classified by cognitive and social-emotional outcome** | | |
|  | Academic performance **(O)** | | |
| Catching |  | Aadland et al. [71] *SES* (3/3) | 0/3 **(0%)** |
| Aiming |  | Aadland et al. [71] *SES* (3/3) | 0/3 **(0%)** |
| Running speed & agility | Jaakkola et al. [90] *SES* (1/24) | Jaakkola et al. [90] *SES* (23/24) | 1/24 **(4.2%)** |
| Leaping | Jaakkola et al. [90] *SES* (14/24)  Jaakkola et al. [90] *MES* (2/24) | Jaakkola et al. [90] *SES* (8/24) | 16/24 **(66.7%)** |
| Motor competence | Syväoja et al. [113] *SES* (2/2) | Chargas [81] *SES* 1/1 | 6/21 **(28.6%)** |
|  | Working memory **(I)** | | |
| Motor competence | Ludyga et al. [97] *MES* (4/8)  Ludyga et al. [97] *LES* (2/8) | Ludyga et al. [97] *MES* (2/8) | 6/8 **(75%)** |
|  | Composite executive functions **(I)** | | |
| Catching |  | Aadland et al. [71] *SES* (3/3) | 0/3 **(0%)** |
| Aiming |  | Aadland et al. [71] *SES* (3/3) | 0/3 **(0%)** |
|  | **Studies classified by motor competence outcome** | | |
|  | Running speed & agility **(I)** | | |
| Academic performance | Jaakkola et al. [90] *SES* (3/8) | Jaakkola et al. [90] *SES* (5/8) | 3/8 **(37.5%)** |
|  | Leaping **(I)** | | |
| Academic performance | Jaakkola et al. [90] *SES* (5/8) | Jaakkola et al. [90] *SES* (3/8) | 5/8 **(62.5%)** |
|  | Motor competence **(I)** | | |
| Academic performance | Syväoja et al. [113] *SES* (1/2) | Syväoja et al. [113] *SES* (1/2) | 1/2 **(50%)** |

*ES* effect size could not be calculated due to lack of information, *LES* large effect size, *MES* moderate effect size, *SES* small effect size

Where adjusted values are used to report significance in studies these are presented

Using the percentage score for each specific association for an outcome (i.e., catching – academic performance, balance – academic performance), the collective influence of these variables on the outcome was collated into a single percentage score and classified as either no association (0–33%), written as ‘0’; indeterminate/inconsistent (34–59%), written as ‘?’; or a positive ‘+’ or negative ‘−’ association (≥ 60%). If there were fewer than three studies in the domain, the strength of evidence was considered insufficient (I) to classify. Any study that included multiple analyses (>8) pertaining to the same path (i.e., object manipulation to working memory) was not included in the results synthesis.

**Electronic Supplementary Table S4.** Analyses and Results (Experimental studies) for pre-school children; studies using causal analyses are highlighted

| **Motor competence and cognitive and social-emotional outcomes** | **Significant causal improvement in IG**  (Reported effect sizes) | **No significant causal improvement in IG**  (Reported effect sizes) | **Summary of results**  **(Analyses reporting a significant improvement/total analyses)** | **Significant aligned improvement in IG**  (Reported effect sizes) | | **No significant aligned improvement in IG**  (Reported effect sizes) | **Significant difference between IG and CG post-intervention** (Reported effect sizes) | **No significant difference between IG and CG post-intervention** (Reported effect sizes) |  |
| --- | --- | --- | --- | --- | --- | --- | --- | --- | --- |
| **Summary of studies classified by motor competence outcome** | | | | | | | | |  |
| Pre-literacy skills, Academic performance, intellectual functioning | | | | | | | | |  |
| Object manipulation |  |  |  | Battaglia et al. [73]^a^  Battaglia et al. [74] |  | | Battaglia et al. [74] *LES* | Battaglia et al. [73]^a^ |  |
| Locomotor skills |  |  |  | Battaglia et al. [73]^a^  Battaglia et al. [74] *LES* |  | | Battaglia et al. [74] *LES* | Battaglia et al. [73] |  |
| Motor competence |  |  |  | Bedard et al. [75] *SES*  Bedard et al. [75] *LES*  Battaglia et al. [74] *LES*  Duncan et al. [86] *LES* | Bedard et al. [75] *SES*  Bedard et al. [75] *MES*  Derman et al. [85] *No Effect* | | Battaglia et al. [74] *LES*  Duncan et al. [86] *LES* | Derman et al. [85] *No Effect* |  |
| Working memory | | | | | | | | |  |
| Object manipulation |  |  |  | Zhang et al. [118]  *LES* |  | |  |  |  |
| Balance |  |  |  | Zhang et al. [118]  *SES-LES* |  | | Zhang et al. [118]  *SES*  Zhang et al. [118]  *SES* |  |  |
| Behavioral regulation | | | | | | | | |  |
| Motor competence |  |  |  |  | Mulvey et al. [101] *MES*  Robinson et al. [109] *MES* | | Mulvey et al. [101] *MES*  Robinson et al. [109] *MES* |  |  |
| Social-emotional skills | | | | | | | | |  |
| Object manipulation |  |  |  |  |  | |  | Minghetti et al. [100] *VSES* |  |
| Locomotor skills |  |  |  |  |  | |  | Minghetti et al. [100] *VSES* |  |
| Motor competence |  |  |  |  | Derman et al. [85] *No effect* | |  | Derman et al. [85] *No effect*  Minghetti et al. [100] *VSES* |  |
| **Summary of studies classified by motor competence outcome** | | | | | | | | |  |
| Object manipulation | | | | | | | | |  |
| Temperament |  |  |  | Taunton et al. [114]  *LES* |  | | Taunton et al. [114] *MES* |  |  |
| Locomotor skills | | | | | | | | |  |
| Temperament |  |  |  | Taunton et al. [114]  *LES* |  | | Taunton et al. [1114] *LES* |  |  |

*CG* Control group*, IG* Intervention group*, LES* large effect size, *MES* moderate effect size, *SES* small effect size, *VSES* very small effect size

*^a^ES* effect size could not be calculated due to lack of information

**Electronic Supplementary Table S5.** Analyses and Results (Experimental studies) for pre-adolescent children; studies using causal analyses are highlighted

| **Motor competence and cognitive and social-emotional outcomes** | **Significant causal improvement in IG**  (Reported effect sizes) | **No significant causal improvement in IG**  (Reported effect sizes) | **Summary of results**  **(Analyses reporting a significant improvement/total analyses)** | **Significant aligned improvement in IG**  (Reported effect sizes) | | **No significant aligned improvement in IG**  (Reported effect sizes) | **Significant difference between IG and CG post-intervention** (Reported effect sizes) | **No significant difference between IG and CG post-intervention** (Reported effect sizes) |  |
| --- | --- | --- | --- | --- | --- | --- | --- | --- | --- |
| **Summary of studies classified by motor competence outcome** | | | | | | | | |  |
| Academic performance, pre-literacy skills, intellectual functioning | | | | | | | | |  |
| Object manipulation |  |  |  | Nobre et al. [103]  *SES-LES*  Nobre et al. [103] *LES* | Nobre et al. [103] VSES-*LES* | |  |  |  |
| Upper limb coordination |  |  |  |  | Botha and Africa [79] *SES* | |  | Botha and Africa [79]^a^ |  |
| Locomotor skills |  |  |  | Nobre et al. [103]  *SES-LES*  Nobre et al. [103] *LES* | Nobre et al. [103] VSES-*LES* | |  |  |  |
| Balance |  |  |  |  | Botha and Africa [79] *MES* | |  | Botha and Africa [79]^a^ |  |
| Running speed & agility |  |  |  |  | Botha and Africa [74] *MES* | |  | Botha and Africa [79]^a^ |  |
| Motor competence |  |  |  | Nobre et al. [103]  *SES-LES*  Nobre et al. [103] *LES* | Nobre et al. [103] *VSES-LES* | |  | Ericsson [87] *MES*  Ericsson [87]^a^ |  |
| Composite executive functions | | | | | | | | |  |
| Balance | **Vazou et al. [117]**  **(2/6 cool executive functions) *MES-LES*** | **Vazou et al. [117]**  **(4/6 cool executive functions) *SES***  **Vazou et al. [104]**  **(hot executive functions) *SES*** | 2/5 **(40%)** | Vazou et al. [117]  *SES-LES* | Katanić et al. [92] *SES* | |  | Katanić et al. [92] *SES* |  |
| Cognitive functioning | | | | | | | | |  |
| Object manipulation |  |  |  | Boat et al. [78]  *SES-VLES*  Magistro et al. [99]  *LES-VLES* | Lee et al. [94]^a^ | |  | Lee et al. [94]^a^ |  |
| Locomotor skills |  |  |  | Boat et al. [78] *SES-MES*  Magistro et al. [99]  *LES-VLES* | Lee et al. [94]^a^ | |  | Lee et al. [94]^a^ |  |
| Balance |  |  |  |  | Katanić et al. [92] *SES* | | Katanić et al. [92] *SES* |  |  |
| Motor competence |  |  |  | Oppici et al. [104] (Low cognitive) *SES-LES*  Boat et al. [78]  *SES-VLES*  Magistro et al. [99]  *LES-VLES* | Lee et al. [94]^a^  Biino et al. [77] *MES*  Oppici et al. [104] (High cognitive) *SES-LES*  Rudd et al. [111] (Creative dance) *SES*  Rudd et al. [111] (Choreography dance) *SES* | | Lee et al. [924^a^ |  |  |
| Creativity | | | | | | | | |  |
| Object manipulation | **Tocci et al. [115]**  ***VSES-VLES*** | **Tocci et al. [115]**  ***VSES-VLES***  **Tocci et al. [115]**  ***VSES-VLES*** | 1/3 **(33%)** | Rodríguez-Negro et al. [110] (GBP) *SES* | Rodríguez-Negro et al. [110] (BIP) *VSES*  Rodríguez-Negro et al. [110] (DLP) *VSES-MES* | |  |  |  |
| Balance |  | **Tocci et al. [115]^a^**  **Tocci et al. [115]^a^**  **Tocci et al. [115]^a^** | 0/3 **(0%)** | Rodríguez-Negro et al. [110] (GBP)  *SES-LES* | Rodríguez-Negro et al. [110] (BIP) SES*-MES*  Rodríguez-Negro et al^.^ [110] (DLP) *SES-MES* | |  |  |  |
| Attention | | | | | | | | |  |
| Object manipulation |  |  |  | Rodríguez-Negro et al. [110] (GBP) *SES-MES* | Pesce et al. [106] *LES*  Pesce et al. [106] *MES*  Rodríguez-Negro et al. [110] (BIP) *VSES-SES*  Rodríguez-Negro et al. [110] (DLP) *VSES-MES* | | Pesce et al. [106] *LES*  Pesce et al. [106] *MES* |  |  |
| Balance |  | **Vazou et al. [117]** | 0/1 **(0%)** | Rodríguez-Negro et al. [110] (GBP) *SES*-LES  Vazou et al. [117] (RG) *SES* | Rodríguez-Negro et al. [110] (BIP) *SES-MES*  Rodríguez-Negro et al. [110] (DLP) *SES-MES*  Pesce et al. [106] *LES* | | Pesce et al. [106] *LES*  Pesce et al. [106] *MES* |  |  |
| Motor competence |  |  |  |  |  | | Ericsson [87]^a^ | Ericsson [87]^a^ |  |
| Working memory | | | | | | | | |  |
| Object manipulation | **Tocci et al. [115] ^a^** |  | 1/1 **(100%)** | Lin et al. [96] (Response accuracy) *MES*  Lin et al. [96]  (Reaction time)  *SES* | Lin et al. [96] (tCNV)^a^ | | Lin et al. [96]  (Response accuracy) *MES*  Lin et al. [96] (iCNV) *MES* | Lin et al. [96] (Reaction time) *SES*  Lin et al. [94] (tCNV)^a^ |  |
| Balance |  | **Tocci et al. [115]^a^** | 0/1 **(0%)** | Lin et al. [96]  (Response accuracy)  *MES-LES*  Lin et al. [96]  (Reaction time) *SES* |  | | Lin et al. [96]  (Response accuracy)  *MES-LES* | Lin et al. [96] (Reaction time) *SES* |  |
| Motor competence | **Oppici et al. [104] *SES-MES*** | **Oppici et al. [104] *MES*** | 1/2 **(50%)** | Koutsandréou et al^.^ [93] *LES*  Rudd et al. [111] (Choreography dance)  *SES-MES*  Oppici et al. [104]  (High cognitive) *SES* | Pesce et al. [106] *SES*  Rudd et al. [111]  (Creative dance) *SES*  Oppici et al. [104]  (Low cognitive) *SES* | | Koutsandréou et al. [93] *SES*  Biino et al. [77] *LES* | Koutsandréou et al. [93] *SES*  Katanić et al. [92] *MES* |  |
| Inhibition | | | | | | | | |  |
| Bal | **Pesce et al. [106]**  ***SES***  **Tocci et al. [115]**  ***MES-VLES*** |  | 2/2 **(100%)** |  |  | | Pesce et al. [106] *SES* |  |  |
| Balance |  | **Tocci et al. [115]^a^** | 0/1 **(0%)** |  |  | | Pesce et al. [106] *SES* |  |  |
| Motor competence |  |  |  | Rudd et al. [111]  (Choreography dance)  *SES-MES* | Biino et al. [77] *SES-LES*  Oppici et al. [104]  (High cognitive) *VSES-LES*  Oppici et al. [104]  (High cognitive) *VSES-LES*  Rudd et al. [111]  (Creative dance) *SES* | |  | Li et al. [95] *VSES-SES* |  |
| Impulse control | | | | | | | | |  |
| Balance |  | **Vazou et al. [117]** | 0/1 **(0%)** |  | Rodríguez-Negro et al. [110] (BIP) *VSES-SES*  Rodríguez-Negro et al. [110] (DLP) *SES*  Rodríguez-Negro et al. [110] (GBP) *VSES-LES*  Vazou et al. [117] *MES-LES* | |  | Rodríguez-Negro et al. [110]  (BIP) V*SES-SES*  Rodríguez-Negro et al. [110] (DLP) *SES*  Rodríguez-Negro et al. [110] (GBP) *VSES-LES*  Vazou et al. [117] *MES-LES* |  |
| Object manipulation |  |  |  |  | Rodríguez-Negro et al. [110] (BIP) *VSES-SES*  Rodríguez-Negro et al. [110] (DLP) *SES*  Rodríguez-Negro et al. [110] (GBP) *VSES-SES* | |  | Rodríguez-Negro et al. [110]  (BIP) *VSES-SES*  Rodríguez-Negro et al. [110] (DLP) *SES*  Rodríguez-Negro et al. [110] (GBP) *VSES-SES* |  |
| Motor competence |  |  |  |  |  | | Ericsson [875]^a^ | Ericsson [87]^a^ |  |
| Behavioral regulation | | | | | | | | |  |
| Motor competence |  |  |  |  | Mulvey et al. [101] *MES* | | Mulvey et al. [101] *MES* |  |  |
| Social-emotional skills | | | | | | | | |  |
| Object manipulation |  |  |  | Jalilnasab et al. [91] *LES* | Berleze and Valentini [76]  *LES-VLES* | | Berleze and Valentini [76] *LES-VLES*  Jalilnasab et al. [91] *LES* |  |  |
| Locomotor skills |  |  |  | Jalilnasab et al. [91] *LES* | Berleze and Valentini [76]  *LES-VLES* | | Berleze and Valentini [76] *LES-VLES*  Jalilnasab et al. [91] *LES* |  |  |
| Balance |  |  |  | Fathirezaie et al. [88]^a^ |  | | Fathirezaie et al. [88]^a^ |  |  |
| Running speed & agility |  |  |  | Fathirezaie et al. [88]^a^ |  | | Fathirezaie et al. [88]^a^ |  |  |
| Motor competence |  |  |  | Jalilnasab et al. [91] *LES* |  | | Jalilnasab et al. [91] *LES* |  |  |
| **Summary of studies classified by motor competence outcome** | | | | | | | | |  |
| Catching | | | | | | | | |  |
| Intellectual functioning |  |  |  |  | De Oliveira et al. [83] *SES* | |  | De Oliveira et al. [83] *SES* |  |
| Balance | | | | | | | | |  |
| Intellectual functioning |  |  |  |  | De Oliveira et al. [83] *SES* | |  | De Oliveira et al. [83] *SES* |  |

*BIP* Balance intervention program, *CG* Control group, *DLP* Drama learning program, *GBP* Game based program, *IG* Intervention group, *PE* physical education, *PEG* Physical education group, *RG* Rhythmic group*, VLES* very large effect size, *LES* large effect size, *MES* moderate effect size, *SES* small effect size, *VSES* very small effect size

*^a^ES* effect size could not be calculated due to lack of information

**Electronic Supplementary Table S6.** Analyses and Results (Experimental studies) for adolescents; studies using causal analyses are highlighted

| **Motor competence and cognitive and social-emotional outcomes** | **Significant causal improvement in IG**  (Reported effect sizes) | **No significant causal improvement in IG**  (Reported effect sizes) | **Summary of results**  **(Analyses reporting a significant improvement/total analyses)** | **Significant aligned improvement in IG**  (Reported effect sizes) | | **No significant aligned improvement in IG**  (Reported effect sizes) | **Significant difference between IG and CG post-intervention** (Reported effect sizes) | **No significant difference between IG and CG post-intervention** (Reported effect sizes) |
| --- | --- | --- | --- | --- | --- | --- | --- | --- |
| **Summary of studies classified by motor competence outcome** | | | | | | | | |
| Composite executive functions | | | | | | | | |
| Motor competence |  |  |  |  |  | | Aadland et al. [72]^a^  Condello et al. [82]  (hot executive functions) *VSES* | Aadland et al. [72]^a^ |
| Cognitive functioning | | | | | | | | |
| Object manipulation |  |  |  |  | Tseng et al. [116] *SES-MES* | |  |  |
| Balance |  |  |  |  | Tseng et al. [116] *SES-MES* | |  |  |
| Working memory | | | | | | | | |
| Motor competence |  |  |  |  | Condello et al. [82] *SES* | |  |  |
| Inhibition | | | | | | | | |
| Motor competence |  |  |  | Condello et al. [82]  *MES* |  | |  |  |
| Social-emotional skills | | | | | | | | |
| Motor competence | **Condello et al. [82] (Enriched PE) *SES*** | **Condello et al. [82]**  ***SES*** | 1/2 **(50%)** | Condello et al. [82]  *SES* | Condello et al. [82]  *SES* | | Condello et al. [82]  *SES* | Condello et al. [82]  *SES* |

*CG* Control group, *IG* Intervention group, *PE* physical education, *MES* moderate effect size, *SES* small effect size, *VSES* very small effect size

*^a^ES* effect size could not be calculated due to lack of information
